# Geometrical Patterning of Super-Hydrophobic Biosensing Transistors Enables Space and Time Resolved Analysis of Biological Mixtures

**DOI:** 10.1038/srep18992

**Published:** 2016-01-12

**Authors:** Francesco Gentile, Lorenzo Ferrara, Marco Villani, Manuele Bettelli, Salvatore Iannotta, Andrea Zappettini, Mario Cesarelli, Enzo Di Fabrizio, Nicola Coppedè

**Affiliations:** 1 Department of Electrical Engineering and Information Technology, University of Naples, 80125, Naples, Italy; 2 Department of Experimental and Clinical Medicine, University of Magna Graecia, 88100 Catanzaro, Italy; 3 Istituto Italiano di Tecnologia, Via Morego 30, 16163 Genova, Italy; 4 IMEM-CNR Parco Area delle Scienze 37/A - 43124 Parma, Italy; 5 King Abdullah University of Science and Technology, Thuwal 23955-6900, Saudi Arabia

## Abstract

PEDOT:PSS is a conductive polymer that can be integrated into last generation Organic Electrochemical Transistor (OECT) devices for biological inspection, identification and analysis. While a variety of reports in literature demonstrated the chemical and biological sensitivity of these devices, still their ability in resolving complex mixtures remains controversial. Similar OECT devices display good time dynamics behavior but lack spatial resolution. In this work, we integrated PEDOT:PSS with patterns of super-hydrophobic pillars in which a finite number of those pillars is independently controlled for site-selective measurement of a solution. We obtained a multifunctional, hierarchical OECT device that bridges the micro- to the nano-scales for specific, combined time and space resolved analysis of the sample. Due to super-hydrophobic surface properties, the biological species in the drop are driven by convection, diffusion, and the externally applied electric field: the balance/unbalance between these forces will cause the molecules to be transported differently within its volume depending on particle size thus realizing a size-selective separation. Within this framework, the separation and identification of two different molecules, namely Cetyl Trimethyl Ammonium Bromid (CTAB) and adrenaline, in a biological mixture have been demonstrated, showing that geometrical control at the micro-nano scale impart unprecedented selectivity to the devices.

Conducting polymers are materials displaying high electrical conductivity, similarly to metal conductors, still maintaining processability, low cost, and the mechanical properties of conventional, commercially available polymers^1^. Discovered in the mid-1970s, they rapidly found application especially in organic electronics, printed electronics, and bioelectronics, in which they are at the basis of a new class of devices with breakthrough properties including easy of fabrication, flexibility and biocompatibility^2^. Organic thin film transistors (OTFTs) are widely explored as sensing devices. The most common OTFT is the organic field effect transistor (OFET), composed of an organic semiconductor film, contacted by the source-drain electrodes, a gate electrode and a dielectric region to provide electrical insulation between the gate and the source-drain channel. Upon biasing the gate, an electrical double layer is formed at the dielectric interface and the current flowing between the source and drain can be modulated according to the field-effect doping^3^,^4^. Differently from OFETs, organic electrochemical transistors (OECTs) are a second generation of OTFT, in which the insulator layer, placed between the channel and gate electrode, is replaced with an electrolyte medium and the conductive polymer is electrochemically active. In the OECTs configuration the conductive polymer is in contact with the electrolyte, in which a gate electrode is immersed. OECTs present unprecedented features including strategic combination with biomedical interfaces^5^, simple and low voltage operation regime^6^ and sensing ability in aqueous environment^7^. A variety of reports in literature demonstrates the sensing efficiency of OECTs in detecting chemical to biological analytes^8^,^9^,^10^ defining OECTs as a new class of biosensor devices, capable of ionic sensitivity. The working mechanism of OECT biosensors relies on the doping/de-doping effect, taking place in the p-type conducting polymer channel: electrolytes within the solution are pushed towards/backwards the conductive polymer because of the DC bias applied at the gate electrode^3^. Such ion-polymer interaction decrease/increases in the hole mobility in the organic semiconductor, modifying the current flowing through the channel. The OECT active layer is commonly made of the conducting polymer poly (3,4-ethylenedioxythiophene) : poly(styrenesulfonate) (PEDOT:PSS), which is a low cost, stable and commercially available material. PEDOT:PSS can be processed into thin films from solution and has a high conductivity^11^. PEDOT is doped by PSS to increases the conductivity of the polymer and also to solve solubility problems^9^. OECT devices based on PEDOT:PSS have been demonstrated in chemical and biological sensing^12^, for controlling cell adhesion^13^ and viability^14^,^15^,^16^, for healthcare monitoring through integration in a natural cotton fiber^12^.

Authors previously presented^17^ a mathematical model that relates the output of an OECT device to the diffusion coefficient (and thus the size) of charged species dispersed in the electrolyte of the device. The transportation of active species in the medium and thus the time of flight (TOF) from an initial position in the electrolyte to the PEDOT:PSS sensing surface have been proved to depend on size and charge of the described species. The TOF, in turn, and the velocity of charge accumulation on the PEDOT surface would determine the time-evolution of the current measured by the OCET device. In comparing experimental results of a real OECT device with the model, we recognized and correctly identified four different species individually placed in a solution. While accurate in determining the diffusion coefficient of individual ions in solution, the described method breaks downs if challenged with complex mixtures. The model is inherently a lumped parameter model and it registers the time signal of the device but does not support spatial resolution. In describing a conventional OECT device, in which the electrolyte is at rest and all convective terms are everywhere vanishingly small in the domain, it reproduces and predicts the arrival events of charge carriers at the PEDOT surface (z coordinate) on time (t coordinate) but it cannot distinguish between different impact point positions (x, y coordinates) on it. The lack of spatial resolution of the device and description thereof results in a loss or degradation of information quantity, quality and density and the inefficacy of the OECT device in resolving a mixture. The separation of multiple species in a solution inevitably requires a spatial separation of the species.

Herein, we propose a micro-patterned PEDOT:PSS conductive layer to realize a super-hydrophobic surface (Fig. 1a) which enables the manipulation and control of fluids at the device interface. The device incorporates five micro-electrodes in a line (Fig. 1b,c), which are the active or sensitive spots in which molecules in a solution are measured. On a similar super-hydrophobic substrate, solutions maintain a spherical shape as suspended in air^9^,^18^,^19^,^20^,^21^,^22^,^23^, and because of its intrinsic curvature the liquid drop develops convective flows within its volume as explained by the celebrated Marangoni effect^24^,^25^,^26^,^27^,^28^,^29^. Convection represents the additional term that may couple to diffusion to allow a time - and space - resolved separation of molecules. Micro-electrodes in the device sense and record the signal variation as a function of time and space and thus correctly identify different species mingled together in a solution (Fig. 1d). The described method extends and completes the design proposed in^9^, where super-hydrophobic PEDOT:PSS pillars are demonstrated with limited sensing capabilities. In the following, we demonstrate the aforementioned device in the sensing of a solution containing both CTAB and epinephrine, such molecules are distinguished with good sensitivity, selectivity and reliability.

## Results and Discussion

### Realizing the Devices

**Figure 2:**
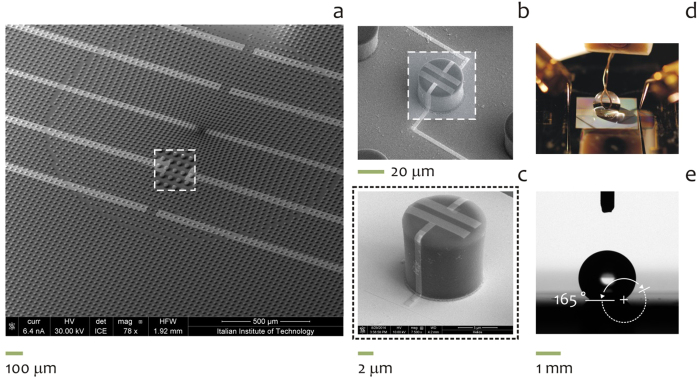
SEM micrograph of the device, the spacing between the micro-pillars in the pattern is not constant while gold-covered circuits run through the pattern of super-hydrophobic pillars (**a**); in the device, a finite number of pillars is modified to incorporate micro-electrodes for site-selective measurement of a solution (**b,c**); the snap-shot in (**d**) shows the device correctly integrated with an external probing station for data acquisition, manipulation and analysis; the hydrophobicity of the system is assured by a fluorocarbon polymer (*C*_4_*F*_8_) deposited on the device, with contact angles approaching 165° **(e)**.

Several SEM micrographs of the devices were recorded over different samples to assess uniformity and reproducibility, and to verify the fabrication process capability to attain extreme control over the key characteristics of the micro-pillars such as shape and size. In Fig. 2a, super-hydrophobic SU8 pillars are positioned on the substrate to form a non-periodic square lattice, in which the distance δ between the pillars smoothly transitions from the center (where the distance is minimum) to the periphery (where the distance is maximum) of the pattern; the function distance obeys here to a function of the sole radius thus exhibiting polar symmetry and this is described in the Methods of the paper and in ref. 30. The described non-periodic tiling is reflected by a non-uniform surface energy density which generates in turn a system of radial forces that recalls the drop to the center of the lattice^30^. The pattern of pillars is intersected by a circuit of five gold conductive tracks, which connect electrodes positioned at the external of the device, to an equally number of pillars in the pattern for space resolved measurements of a solution (Fig. 2b). The described pillars are modified to incorporate micro-electrodes as in Fig. 2c and represent the active or sensitive spots of the device. The electrodes are here combined in a system of two opposite facing parallelepipeds, where the distance between the parallelepipeds is in the low micrometer range, and thus an externally applied voltage would generate large and confined (strongly localized) electric fields. The entire system is coated with a conductive PEDOT:PSS polymer (see Methods) and the integration of a similar material with the described micro-scale geometry enables to measure the electric activity of ionic species in a solution as a function of time and space. An image of the device is reported in the inset of Fig. 2d correctly integrated with an external probing station for data acquisition, manipulation and analysis. The hydrophobicity of the system is assured by a fluorocarbon polymer (C_4_F_8_) deposited on the device: here surface chemistry and geometry combine^20^ to realize a device with an increased hydrophobicity and contact angles as large as 165° (Fig. 2d). The convective flows that develop within the drop shall enable the separation of different species in a solution, and this is described in the following of the paper.

### Saline Electrolyte local concentration dependence in a single drop

**Figure 3:**
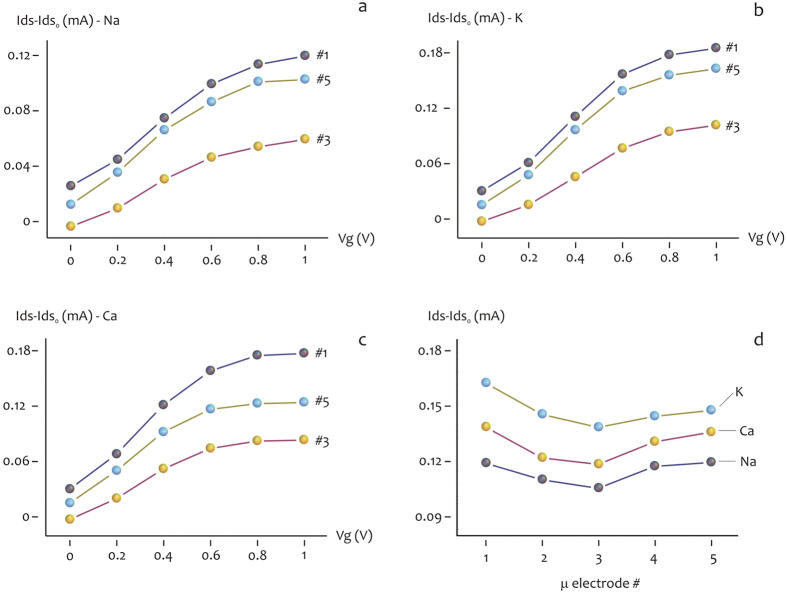
Device response for saline solution of NaCl, KCl and CaCl 10^−3^ M. Response for device at the border #1,#5 and at the center #3 for NaCl (**a**), KCl (**b**), CaCl (**c**). In (**d**) a comparison of the response for a fixed Vg = 0.8 V for the three saline solution respect to the spatial position of the devices.

To have a clear and systematic characterization on the local topographic properties of the device and hence of the analytes in the drop, three different saline electrolytes have been analyzed in five different topographic position from the periphery (#1) to the center (#3), repeatedly to the periphery (#5) of the substrate. The OECT modulation measurements have been performed at 10^−3^ M concentration for each saline electrolyte NaCl, KCl and CaCl. The transient response are reported in Supporting Information Figures #1.1 and #1.2, representing the Ids current at different Vg incrasing from 0 to 1 V with 0.2 V steps. Each measurement has been performed on every single device from one side to the other, with respect to the drop position. The absolute value of the current for each device results higher for the device at the border (#1–#5), it decreases for intermediate (#2–#4) and finally is the lowest for the central device (#3). Figure 3a–c report the absolute value of the output curves in presence of a gate potential ranging from 0 to 1 V. Again, the current modulation is higher for the device at the border, intermediate for device #2–#4 and lower for the central device #3. In Fig. 3d The three saline species are compared reporting the relative modulation at a fixed Vg = 0.8 V in function spatial position of the device, from the center to the border of the drop. The relative variation in current is related to the concentration of positive ions in the device. The data present a coherent variation that shows a higher variation of the Ids current for the border respect to the center. Moreover the increase is present from both direction moving to the borders from the center. This is ascribed to a variation of the ions concentration in the drop and not to statistic difference of the devices. The ionic species present either different charge (being *z* = 1 for sodium and potassium and *z* = 2 for calcium) and different diffusion coefficients, ranging from *D* = 0.706 × 10^−9^ *m*^2^/*s* for calcium to *D* = 1.334 × 10^−9^ *m*^2^/*s* and *D* = 1.96 × 10^−9^ *m*^2^/*s*, for sodium and potassium. This translates into different characteristic time response for I_ds_ current of the salts (Supporting Information Figure #1.3): the transient behavior is different for the three species reflecting the properties of different diffusion for the potassium respect to sodium and the different charge of Calcium, that allows the highest current variation. Notice that Ca, K, and Na are ordered for decreasing intensity in Supporting Information Figure #1.3. This is consistent with the fact that Ca has a charge number *z* = 2, and present a comparable diffusion coefficient, while the different behaviors of K and Na in modulation dynamic, with a lower evolution in time, could be related to their *z* = 1 charge number. Among K and Na a different kinetic behavior of the transient corresponds to a different diffusion coefficient: K presents higher transient, corresponding to a higher diffusion coefficient (*D* = 1.96 × 10^−9^ *m*^2^/*s*), while Na (*D* = 1.334 × 10^−9^ *m*^2^/*s*), which presents lower intensity transient, has a lower diffusion coefficient. Among the differences in the kinetics of the current variation, each saline electrolyte reflect the behavior of a higher concentration in the borders and a lower concentration in the center. The species with a higher diffusion and higher charge as potassium and calcium reduces the difference between center an border, while sodium, with a reduced diffusivity present a higher topographic difference.

### Measuring individual biological species in a solution: the space dependency

**Figure 4:**
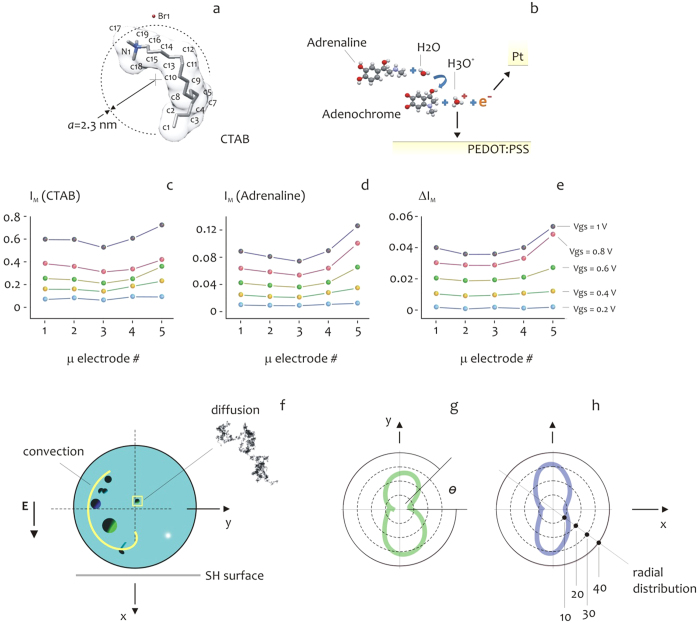
Plain-stick representation of a CTAB molecule with an hydro-dynamic radius *a*^*CTAB*^ ~ 2.3 *nm* (a). A schematic of the electro-oxidation reaction of adrenaline in a solution: in water, adrenaline is separated into adrenaline-quinone and adreno-chrome at the surface of the gate electrode of platinum, from this an ion of hydrogen *H*^+^ is released that immediately forms the super-molecular complex *H*_3_*O*^+^ (**b**). The modulation current *I*_*M*_, that is, the maximum drain-source measured on a time interval, is reported for CTAB (**c**) and adrenaline (**d**) as a function of position on the device and gate voltage *V*_*gs*_. For both molecules, the intensity of current is larger at the border of the device compared to the center. The difference of current between adrenaline and CTAB (**e**) reveals that adrenaline is transported to the periphery of the pattern with an increased efficiency compared to CTAB: the discrepancy between adrenaline and CTAB may be ascribed to the sole difference in mass between those species. The scheme in (**f**) depicts the main drivers of transportation for a solute trace in a drop: the experimental conditions generate within the drop Marangoni convective flows due to the curvature of the drop, which combine with diffusion and the external applied electric field and these are the forces exerted on the particle in solution. The balance/unbalance between these forces is dependent on particle size and would ultimately determine the field of motion of the particle within the drop. The simulated radial distribution of an analytical trace after 3 s from deposition, where the size of the particles constituting the trace has been varied from *a* = 0.02 nm (**g**) to *a* = 2 *nm* (**h**). Smaller particles distribute preferentially laterally in the drop differently from larger particles.

The procedure described above has been used to measure and analyze biological species individually placed in a drop and deposited on the device. These are (i) cetyl trimethylammonium bromide (CTAB), and (ii) Epinephrine, that is commonly known as adrenaline. CTAB and adrenaline were used here in a concentration of *C* = 10^−4^ *M*. CTAB is a cationic surfactant^12^, that is, an organic molecule in which its hydrophilic head group carries a positive net charge, and thus *z* = 1. The concentration used here is below the critical concentration at which CTAB monomers cluster together to form supramolecular aggregates of those monomers^12^ (micelles), and thus we shall consider here CTAB molecules individually stabilized by the solvent, hence the molecular weight is 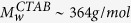 and the diffusion coefficient^31^ is *D*^*CTAB*^ ~ 10^−10^ *m*^2^/*s*, from which the Stokes radius *a*^*CTAB*^ is estimated^32^ being 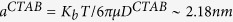. In deriving *a*^*CTAB*^, we considered the viscosity of water *μ* = 10^−3^ *Pas* at the room temperature *T* = 289 *K*; while *K*_*b*_ ~ 1.38010^−23^ *J*/*K* is the Boltzmann constant. The structure of CTAB is reported in Fig. 4a.

Adrenaline is a functional neurotransmitter, playing a central role in many instinctive responses, especially under stress situations and strongly physical strengthens. A timely sensing of abnormal adrenaline concentration could be a fingerprint of a pathological situation, like panic or heart attack, or could identify a typical flight, fight and fright response^33^. The sensing mechanism of adrenaline using the OECT device is somewhat more complex than CTAB and it is explained as follows: adrenaline undergoes an electro-oxidation resulting in adrenaline-quinone and adreno-chrome formation at the surface of the platinum gate electrode, releasing *H*_3_*O*^+^ ions into the solution (Fig. 4b). This generates, in turn, a faradaic current that flows in the source-gate circuit and represents the timely signal measured by the OECT device. An thus, in measuring adrenaline, we should actually consider the cationic complex *H*_3_*O*^+^ for which^34^ *z* = 1, 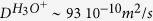 and thus 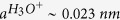. In what follows, we shall thus compare the response of a molecule to that of hydronium ion, where the difference in size between the two spans near two orders of magnitude, while the charge number is fixed. The maximum drain-source CTAB current *I*_*M*_ measured on a time interval as a function of micro electrode number and thus position on the device is reported in Fig. 4c; the values are presented for different values of gate voltage *V*_*gs*_ and are renormalized with respect to the base or unperturbed current *I*_*dso*_. On analyzing the diagrams, one may observe that the modulation current *I*_*M*_ smoothly increases on changing *V*_*gs*_ from *V*_*gs*_ = 0.2 *V* to *V*_*gs*_ = 1 *V*, more important than this, the device measures *I*_*M*_ currents that depend on the position of the active micro-electrode on the substrate, and are generally lower at the center and larger at the border of the drop. Observing that the current measured by the OECT is generally proportional to the number of charge carriers that are transported to the conductive polymer sensitive region over time^17^, the diagrams in Fig. 4c indicate that CTAB molecules are prevalently carried to the periphery of the device (and the of the contact line of drop), and this effect is amplified by the voltage at the gate *V*_*gs*_. Also note that the symmetry of *I*_*M*_ for all the considered *V*_*gs*_, reflects the polar symmetry of the device. The diagrams in Fig. 4d are similar to those presented in Fig. 4c, except that they were derived for adrenaline. While the general trend of *I*_*M*_ as a function of space and voltage is preserved, and thus the comments considered for CTAB may be equivalently applied to adrenaline, one can note that the dependency of *I*_*M*_ on micro electrode number is, for this configuration, more pronounced. For the particular case of the modulation measured at a fixed *V*_*gs*_ = 0.8 *V*, considered as an example, moving from the center (#3) to the periphery (#5) of the device, the adrenaline current nearly doubles its value, passing from *I*_*M*_ ~ 0.05 ~ 0.05 to *I*_*M*_ ~ 0.10. The difference in current 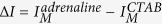 between the two species is presented in a separate Fig. 4e, where it is clear the increased propensity of adrenaline to target and thus distribute to the boundary of the device, compared to CTAB. Recalling what it has been said in the above, considering that adrenaline (that is, *H*_3_*O*^+^) and CTAB have a similar charge number *z* = 1 and that all the remaining experimental conditions across the measurements have been maintained unvaried, the discrepancy between adrenaline and CTAB as from Fig. 4c–e may be ascribed to the sole difference in mass between those species.

Consider the scheme in Fig. 4f for ease of visualization. A solution, deposited on the micro-structured/super-hydrophobic substrate, maintains a quasi-spherical shape, this is responsible for Marangoni convective flows which develop within the drop^17^,^24^,^25^,^29^ with streamlines which vary from straight (at the center) to highly curved (at the periphery of the drop), and this is explained in the Methods of the article and in a separate Supporting Information file. An electric active particle (an ion) in the drop would be thus transported over time driven by convection, diffusion, and the externally applied electric field^9^,^17^: the balance/unbalance between these forces will cause the trace to be transported to specific regions of the drop and thus to deposit differently on the device. Observing that, recalling the celebrated Einstein relation, the diffusion coefficient *D* of a particle is inversely proportional to its radius *a*


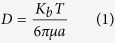


while, the Stoke’s drag force *F*_*drag*_ (that is, the convective term) exerted on the same particle is directly proportional^32^,^35^ to *a* (in what follows, *u* is the magnitude of the velocity vector field in a point)


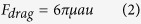


for a fixed *z* number, the net forces that are exerted on a particle and the particle distribution itself in a drop (and thus in close proximity of the substrate) will depend on the size of the particle. In Fig. 4g,h, we present for comparison the simulated radial distribution of an analytical trace after 3 s from deposition, where the size of the particles constituting the trace has been varied from *a* = 0.02 nm (Fig. 4g) to *a* = 2 *nm* (Fig. 4h), as to reproduce the experimental conditions. The distributions have been obtained on solving numerically the Langevin equation^36^, which describes the motion of a particle exposed to diffusion, convection, and electric forces in a domain (Methods), for *n* = 500 initial particles randomly distributed in the drop. The numerical solution confirms that smaller particles distribute preferentially laterally in the drop (that is, at the periphery of the device), differently, larger particles are preferentially transported in close proximity of the device, in line with the experimental evidence. In this section, we considered different species separately placed in a solution, and demonstrated the spatial localization of a solute that depends on the size of the particles of the solute. This concept may be extended to species considered together in a solution (biological mixtures) that can be thus resolved, and this is demonstrated in the remaining of the Results.

### Measuring individual biological species in a solution: the time dependency

**Figure 5:**
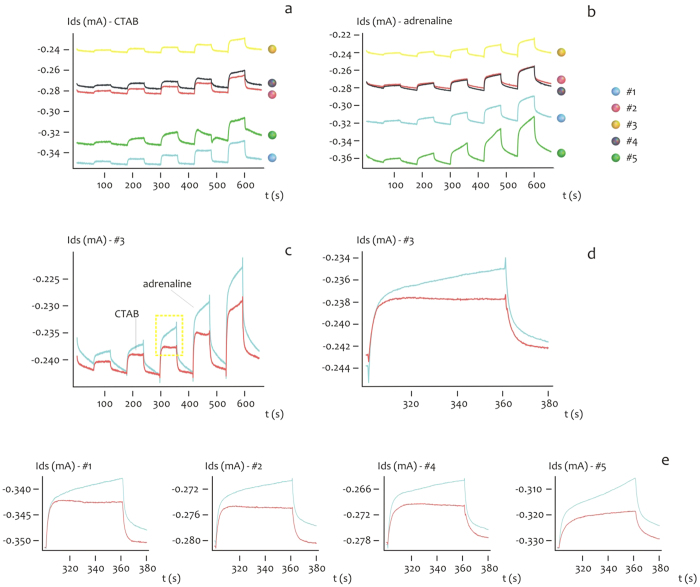
The time evolution of the drain current *I*_*ds*_ for CTAB (**a**) and Adrenaline (**b**) for a fixed *V*_*gs*_ = 0.8 *V*.A direct comparison between CTAB and adrenaline is presented in (**c**) for the micro electrode #3, that is, the center of the device. The inset in (**d**) unveils the different time dynamics between CTAB and adrenaline for a specific time interval 300–380 *s*. For CTAB, the micro sensor behaves as if the output conforms to the standard curve of a second-order response. Differently, when considering adrenaline, the time-dependent characteristic of the system resembles that of a first order system. The described trend is generally repeated for all the micro-electrodes from #1 to #5 in line on the device (**e**).

**Figure 6:**
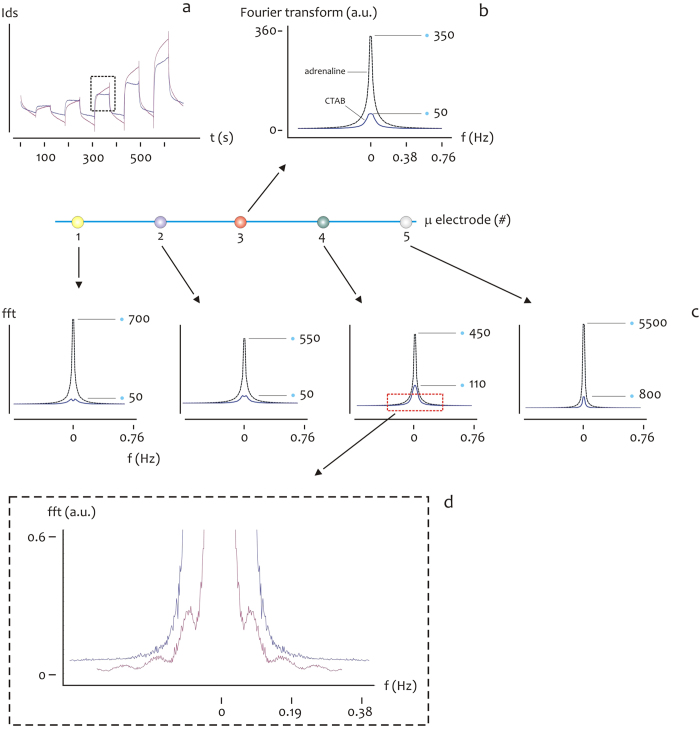
The characteristic time behavior of CTAB and adrenaline and the difference between these may be ascribed to the different size and thus motility of the considered species in a solution, and is reflected by the Fourier transform of the time signal of CTAB compared to adrenaline. The square of the real part of the Fourier transform of CTAB and adrenaline (**b**) is reported, derived from the time-continuous signal of the two species for time interval 300–380 *s*, for a fixed *V*_*gs*_ = 0.8 *V* (**a**). The Fourier transform of adrenaline express peaks (maximum fft amplitudes) that are higher than those displayed by CTAB, for all the considered points in the device, that is, from #1 to #5 (**c**). On magnifying the small frequencies region as in in (**d**,**e**) one may also observe that the Fourier profile of CTAB is more rough and discontinuous compared to adrenaline.

The time evolution of the drain current *I*_*ds*_ for CTAB and Adrenaline is reported in Fig. 5a,b, for a fixed *V*_*gs*_ = 0.8 *V*. Differently from the above, the values of current are presented as rough data (that is, they are not renormalized) from which one can notice at a glance the minimum of current measured at the center of the substrate (micro electrode #3), and progressively increasing moving from the center to the periphery (#2, #4), to the border of the device (#1, #5), where the currents are considered here in their absolute values. A direct comparison between CTAB and adrenaline is presented Fig. 5c for the micro electrode #3 and in the inset in Fig. 5d for a specific time interval, from which one may observe the different dynamics expressed by the species. For CTAB, the micro sensor behaves as if the output conforms to the standard curve of a second-order response, here, the response is faster with the typical overshoot of similar dynamic systems, where the rise time *t*_*r*_ and the time constant *τ* are vanishingly small, *t*_*r*_→0, *τ*→0, where *t*_*r*_ is here defined as the time required for the response to rise from 10% to 90% of its final value, and *τ* is the time which is required by the sensor to reach 90% of the steady-state, upon application of the external voltage. Differently, when considering adrenaline, the time-dependent characteristic of the system resembles that of a first order system, here, the response is slower compared to the output curve of CTAB, and *t*_*r*_ and *τ* are generally different from zero. Notice also that adrenaline rises to values of current (in modulus) that are generally higher than CTAB, and this may be explained considering the larger mass of CTAB compared to that of adrenaline: CTAB molecules, transported to the PEDOT:PSS sensing surface, would more easily occupy the free sites of the surface and thus saturate the surface itself, forming an hydrophobic shield which prevents the transportation of additional charge carriers to the device and the de-dope mechanism of PEDOT:PSS. The time characteristics of the *I*_*ds*_ current and differences thereof observed for CTAB and adrenaline at the micro electrode #3 is generally repeated at the remaining micro electrodes from #1 to #5, and this is reported in Fig. 5e. The characteristic time behavior of CTAB and adrenaline and the difference between these may be ascribed to the different size and thus motility of the considered species in a solution^37^,^38^,^39^, and is reflected by the Fourier transform of the time signal of CTAB compared to adrenaline. Consider the diagrams in Fig. 6. Here, we report the square of the real part of the Fourier transform of the signals (Fig. 6a) considered in the same time interval 420–480 s, for a fixed *V*_*gs*_ = 0.8 *V*. This represents the information content of the current trace at different frequencies. The Fourier transform of adrenaline express peaks (maximum fft amplitudes) that are higher than those displayed by CTAB, for all the considered points in the device, that is, from #1 to #5 (Fig. 6b,c). On magnifying the small frequencies region as in the inset in Fig. 6d,e, one may also observe that the Fourier profile of CTAB is more rough and discontinuous compared to adrenaline, that is, CTAB presents more peaks than adrenaline. This is easily explained considering that the CTAB response resembles a square pulse, that excites more frequencies than a smooth continuous function.

### Measuring CTAB and adrenaline in a mixture

**Figure 7:**
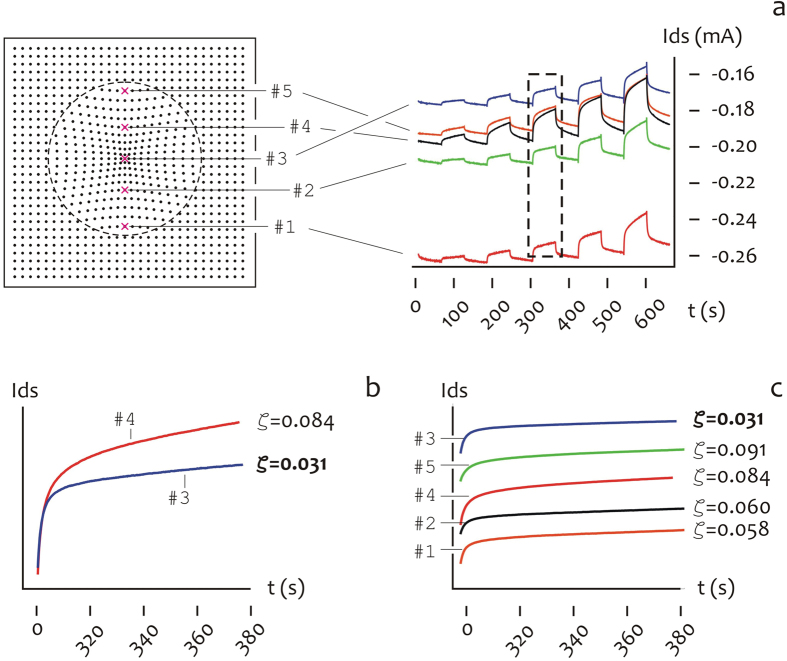
Time traces of an isomolar solution of CTAB and adrenaline (*C* = 10^−4^ *M*) measured for all the PEDOT/conductive micro electrodes in a line on the device, from #1 to #5 (a). The inset in (**b**) magnifies the time curve of the current in the 300–380 *s* time interval: from this one may observe that derivative of the signal in #3 is small, 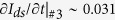, compared to that measured in #4, 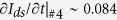, that nearly triples the value in #3. This is generally observed over the entire surface of the device in which the derivative of the time signal smoothly transitions from the center to the periphery of the pattern of super-hydrophobic pillars (**c**).

**Figure 8:**
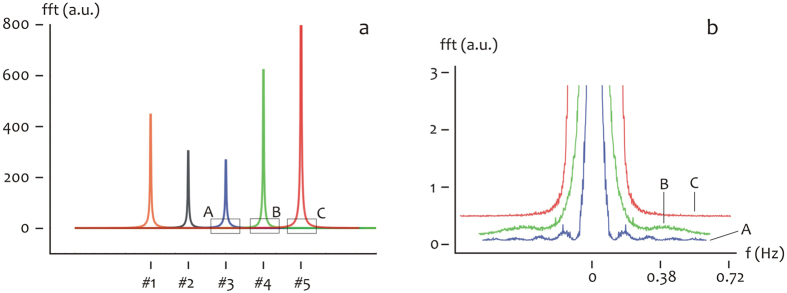
The Fourier transform of the CTAB + adrenaline mixture time signal is reported in (a) as a function of micro-electrode number. The amplitude of the Fourier transforms decreases moving from micro electrode #1 to micro electrode #3, where it attains a minim. Then, it rapidly increases for increasing micro-electrode number. The inset in (**b**) compares Fourier transform of the time signal in the low frequency limit for points from #3 to #5. At the center of the device (#3), rather than at the periphery (#4), the fft profile of the solution displays numerous peaks.

While CTAB and adrenaline have been analyzed heretofore in isolation, here a mixture of equimolar solutions of CTAB and adrenaline is instead considered, where the initial concentration for the species is *C* = 10^−4^ *M*. The time signal of the mixture is reported in Fig. 7a measured for all the PEDOT/conductive micro electrodes in a line on the device, from the periphery (#1) to the center (#3), repeatedly to the periphery (#5) of the substrate. The *I*_*ds*_ currents are reported in Fig. 7a as rough data, and thus the current measured in #3 (that is, the center of the device) is small (in modulus) compared to the current measured in #1 (that is, the border of the device), and this confirms the previously observed fact that species in a solution are preferentially transported to the external of the drop. More important than the absolute values of the *I*_*ds*_ current, the time derivative of the current itself may provide exclusive (otherwise unattainable) information about the relative abundance of CTAB and adrenaline on the device. Consider the inset in Fig. 7a, magnified as in Fig. 7b for the time signal measured in #3 compared to the signal measured in #4, where the analysis is restricted to the 420–480 s time interval for mathematical convenience. The derivative of the signal in #3 is small, 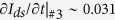, compared to that measured in #4, 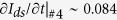, that nearly triples the value in #3. Recalling that the time response of pure CTAB resembles a flat response typical of a second order system, compared to pure adrenaline that instead reproduces the linear response of a first order system, the diagrams in Fig. 7b suggest that the relative abundance of CTAB to adrenaline in a solution experiences a threefold increase moving from #4 to #3. This is generally observed over the entire surface of the device in which the derivative of the time signal smoothly transitions from the center to the periphery of the pattern of super-hydrophobic pillars (Fig. 7c). Pushing further our thesis, assuming linearity, whereby a linear superposition of simpler effects may reproduce the response of the mixture, the relative abundance of CTAB and adrenaline *χ*_*CTAB*_, *χ*_*adrenaline*_ may be derived everywhere on the substrate


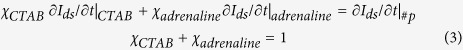


where 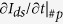 is the current measured on a generic position *p* on the device, while 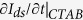 and 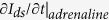 are the derivatives of the time traces of sole CTAB and sole adrenaline as presented in the paragraph §2.4. Using Eq. (3), we obtain 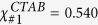, 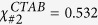, 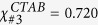, 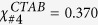, 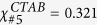, and thus CTAB accumulates at the internal of the device differently from adrenaline. Consider for consistency the Fourier transform of the mixture signal as a function of micro-electrode number as in Fig. 8a. The amplitude of the Fourier transforms decreases moving from micro electrode #1 to micro electrode #3, where it attains a minimum. Then, it rapidly increases for increasing micro-electrode number. And thus the Fourier transform amplitude recovers an inflation rapidly followed by a deflation. Considering the diagrams in Fig. 6a,b, where the Fourier transform of CTAB overruns the Fourier transform of adrenaline from 5 to more than 10 times, the findings presented in Fig. 8a and in Fig. 7 are in qualitative agreement, and both support the evidence that CTAB molecules are spatially separated from adrenaline on the device. This is also evident from the inset in Fig. 8b, where the Fourier transform of the signal is presented in the low frequency limit for points from #3 to #5, and here one may notice peaks gradually emerging from the Fourier transform profile, moving from point #5 where the percentage of CTAB is low, to point #3, where the percentage of CTAB is larger and a wide spectrum of frequencies is consequently excited. The observation that different molecules in a solution present different Fourier transform profiles (that are, the characteristic dactylogram of those molecules) may be at the basis of an identification procedure in OECT devices, similar in concept to the peak assignment method realized in Raman or other spectroscopies, in which the contributions of individual molecules in a mixture are resolved in a complex spectrum^40^,^41^,^42^,^43^,^44^. The time evolution of the current of a charged specie in solution represents a trove of information reflecting the specie’s charge and size and is for the time being limited to few applications. Previously^17^, we demonstrated that the charge *q* and the diffusion *D* of an ion in an electrolyte at rest (that is, the convective terms are vanishingly small everywhere in the domain) may affect the output of an OECT sensor and discussed the results in relation of the label free analysis of the analytical content of a solution in which different species were individually placed. Herein, we developed a more sophisticated evolution of the method. We modified a conductive PEDOT:PSS polymer to include extra non-continuous scales. These imparts to the OCET device improved functions and properties including an augmented hydrophobicity and the ability to manipulate biological solutions. A solution, deposited on the substrate, would maintain a quasi-shape and the curvature at the liquid-air interface would generate, in turn, convective Marangoni flows within the drop. While diffusion has effects on the measured signal on time, convection has the complementary effect of changing the trajectories of molecules in the drop depending on molecule size, and thus it would influence the space dependence of the solution.

### Remarks on possible applications of the device in clinics, research and industry

This paper presents a proof of concept whereby species with different sizes, charges, or both, can be separated in a mixture, and the separation is operated by the competition between diffusion and convection in a drop. One ingredient of a similar separation is therefore the drop itself and the physics behind it. Another ingredient, is a thorough analysis of the signal acquired over time over different sites of the device, that has here been realized using (also) Fourier based variables including the Fourier transform of the signal. Here, we limited the analysis to an ideal mixture of two species in which the sizes of the species differ to a great extent and the charge is maintained constant. Clearly, a proof of concept necessitates an effective demonstration of a process operated in conditions in which the completion of the process (here, the separation of species) is understandable, possibly simple, certainly unequivocal. Current practice in medicine and analytical chemistry may be very different. In real applications, the device would be challenged with mixtures in which the components of the mixture are many in number and the size and charge of those components are distributed over perhaps narrow intervals. Examples may include the identification and detection of specific biological markers in samples extracted from serum or other biological fluids, to evaluate or predict the incidence of outcome or disease, or the susceptibility of the disease to a given treatment: the separation and identification of the content of similar waste deposits, and the unsupervised classification of this content into defined groups with a small error may be used for the evaluation of the health status of patients even by not trained or minimally trained personnel, or for exploratory data analysis to find hidden patterns in data reflecting the ongoing patient assessment, with major beneficial consequences for the national health care. In this and other similar examples, in which multiple species populate a solution, the identification of those species is still possible provided that the number of active sites of the device is sufficiently large to equal the number of the species of interest (targets) in that solution. Assuming linearity, the response on a site is given by the superposition of the signals of individual species. If we have *n* species, we shall have *n* unknowns which are the concentrations of those species in solution. We may find a solution, if we can write the same number of equations, where each equation describes the time evolution of the signal acquired at a specific measurement point. Under these conditions, we say that the system is determinate. However, a determinate system is not the sole requirement for retrieving the solute concentrations. We also need an *a priori* knowledge of how individual ions behave. Therefore, operatively, a similar analysis should be accompanied by the realization of (perhaps massive) data bases, made available on line, in which specific time traces are associated to specific biological species (that is, the structure of relevant analytes is encoded in terms of OECT signals). From the cross comparison of a similar data base with experiments, the composition of a solution would be determined. A preliminary purification of the biological sample by ultra-centrifugation may perhaps facilitate this identification. Another concern for the described device is *safety*. As it stands, the device operates in an environment in which the personnel, the patient, or both, are exposed to the chemical/biological hazards of the drops, and these may be effective in situations in which the drop is extracted from cancer patients or patients with infectious diseases. Moreover, the device may be used repeatedly for multiple measurements without loss of functionality, which increases the probability of contamination and associated risks for the operator. To eliminate or reduce these risks, two strategies are here proposed.

In the first scenario, the device is operated by highly trained personnel, this, however, would increase the costs of the analysis exceeding those of conventional screening/diagnosis techniques.

In the second scenario, the super-hydrophobic substrate is integrated in a micro-fluidic pneumatic chamber, in which the walls of the chamber prevent direct contact of the inner volume with the human operator, similarly in concept to a biological safety cabinet in which bio-contaminants are maintained under control. The deposition of the drop would be realized using an automatic, a semi-automatic, or a manual dispenser, moreover, the interface between the internal and external of the device should be designed to guarantee that the exposition to the biological sample is prevented at any time. A similar device would be used an unlimited number of times for the same sample, then it may be either (i) cleaned and sterilized (using repeatedly solution free DI water droplets and UV irradiation), or (ii) disposed. The latter option would be still cost effective: considering that the fabrication of the device makes use of (conventional) techniques derived from the microelectronics industry (for example, photolithography), large scale production of a similar device would dramatically abate its fabrication costs.

## Conclusions

We micro-patterned the conductive PEDOT:PSS polymer to obtain a super-hydrophobic OECT device in which a finite number of points on the device is independently controlled for site-selective measurement of biological species in a solution. We demonstrated how the combination of convection and diffusion (and thus, time and space effects) may be used to separate biological species in a solution on a single super-hydrophobic OECT device, with high sensitivity, selectivity and reliability. The extra non-continuous scales result in vanishingly small friction forces on the surface and thus a solution on in it would maintain a spherical shape as suspended in air. Due to its curvature, Marangoni convective flows develop within the volume of a drop of solution. The competition between convection and diffusion will cause a spatial separation of biological species that would depend on the size and charge of the species in a solution. On realizing a time and space resolved measurement of the solution, the described OECT device may operate the identification and separation of different species mingled up in a solution with high sensitivity, selectivity and reliability. We used this strategy to distinguish CTAB from adrenaline molecules in a binary mixture.

## Methods

### Fabrication of the devices

Devices sensitive to charged species in a solution were designed and micro fabricated. The devices are given by the superposition and correct alignment of different layers. Layer A, that is a silicon substrate which contains conductive gold circuits connecting the electric active areas in the interior of the device, to the metal contacts (source and drain electrodes) positioned at the border of the device for experimental convenience. Layer B, which comprises super-hydrophobic SU8 micro pillars arranged to form a square pattern, in which the spacing of the pillars is not constant over the domain and this permits the automatic overlap of the solution droplet with the center of the device. A certain number of pillars (here, 5) are further modified to incorporate micro-electrodes, and thus the final devices contain discrete active spots or points that smoothly transition from the center (point #3) to the border (point #1, #5) of the droplet. In doing so, the device may measure the electric activity of active species in specific points of the solution and this can be space-resolved. The entire substrate is therefore spin coated with a conductive PEDOT:PSS thin film. A fluorocarbon polymer (C_4_F_8_) is finally deposited on the devices which, on account of their hierarchical structures bridging different length scales, exhibit an increased hydrophobicity with contact angles as large as nearly 170°. An artistic representation of the device is reported in a separate Supporting Information file #3.1. The cartoon describing the entire device fabrication process is reported in a separate Supporting Information file #3.3.

#### Microfabrication of gold conductive patterns on the supporting silicon substrates (Layer A)

P-doped, (100) silicon wafers with resistivity of 5–10 Ohm/cm were used as substrates; they were cleaned with acetone and isopropanol to remove possible contaminants and then etched with a 4% hydrofluoric acid (HF) solution. The wafers were then rinsed with DI water and dried with N_2_. Standard optical lithography techniques (Suss Microtec MA6/BA6, Sunnyvale, CA, USA) were employed to generate patterns of the circuits within a layer of positive tone resist (S1813) that was spin-coated onto clean silicon wafers. The masks necessary for optical lithography were fabricated using direct SEM (Scanning Electron Microscope) lithography as described in a separate Supporting Information file #4. Upon evaporation of a 70 nm layer of gold on the sample, a lift-off process in an excesses of acetone was used to remove the un-exposed resist from the substrate, and define the pattern of gold circuits.

#### Microfabrication of Patterned Super-hydrophobic Surfaces (Layer B)

A non-periodic pattern of super-hydrophobic micro pillars was realized on the substrate. Following the methods described in^30^, an original square pattern of pillars was modified to obtain a lattice in which the distance *δ* between adjacent pillars smoothly transitions from the border (*δ* = 30 *μm*) to the center (*δ* ~ 11.2 *μm*) of the lattice, according to the power law *δ* = *H**r**f* (*δ*^*o*^), where the superscript *o* stands for original, ***r*** is the polar coordinate that indicates the position of a pillar from the origin or center of the pattern *O*, H = 0.89 is a positive constant, and *f* (#) = 0.1 + (#/*f*)^*ε*^, with *ε* = 0.35, and the length of the variable region in the pattern *l* = 1350 μm. The diameter of the pillars is set as d = 10 μm while the height is h = 20 μm. In doing so, a variable tiling of the surface is obtained, which in turn translates into a non-uniform surface energy distribution, with a minimum of energy at the center of the pattern *O*, and this guarantees that a drop would be automatically centered in *O* upon deposition. P-doped, (100) silicon wafers with resistivity of 5–10 Ohm/cm were used as substrates; they were cleaned with acetone and isopropanol to remove possible contaminants and then etched with a 4% hydrofluoric acid (HF) solution. The wafers were then rinsed with DI water and dried with N_2_. Standard optical lithography techniques were employed to generate the described pattern of pillars using the negative tone resist SU8-25, that was spin-coated onto clean silicon wafers. In this case, the masks necessary for optical lithography were fabricated using direct laser writing (Heidelberg DWL 66 fs), with the final design processed reported in a separate Supporting Information file #4.3, 4.4. The correct alignment of layer A with layer B was assured by alignment markers conveniently positioned at the margins of both patterns.

#### Realizing Micro-Electrodes on the Top of the Pillars

Some pillars were further modified to incorporate metallic contacts which connect the described pillars to the gold contact area. This task was performed using EBID (Electron Beam-Induced Deposition) fabrication. This process consists in injecting a precursor gas including Pt-C into the SEM chamber; the gaseous molecules are then hit by the electron beam and precipitate onto the substrate with a high spatial accuracy. For each pillar, the platinum deposition comes into three steps. In the first step, the contacts are deposited on top of the pillars. A SEM column current of 1.6 nA and voltage 20 kV are set and these permit to deposit a layer of platinum approximately 100 nm thick and the remaining dimensions specified in a separate Supporting Information file #4.2. In the process, a dwell time of 200 ns and a thickness parameter of 15 μm are set. In doing so, horizontal lines are realized which protrude from the pillar, creating a deposition on the bottom useful for a later connection to the gold structure. The second step consists in the connection to the gold track. Since the distance between lines ranges from 60 to 70 μm, the process of fabrication becomes considerably time consuming. To overcome this limitation, we increased the electronic current and voltage to 6.4 nA and 30 kV, respectively. In the last step, we realized the connection on the side of the pillar. For doing so, we turned and tilted the substrate to an angle of 45° in order to deposit on the lateral surface of the pillar.

#### The Conductive PEDOT:PSS Thin Film

A solution of poly(3,4-ethylenedioxythiophene) doped with poly (styrene sulfonate), PEDOT:PSS (H.C. StarckClevios PH500), was spun onto the silicon substrate with pillars substrate so that an estimated film thickness *d* ∼ 80 nm was achieved. The PEDOT:PSS solution was previously doped with ethylene glycol (Sigma Aldrich) to enhance its electrical conductivity and dodecyl benzene sulfonic acid (DBSA) surfactant (Sigma Aldrich) to improve film forming. After the spinning the device was baked on a hotplate at 140 °C for 60 min. The substrates were then covered with a thin (few nm) film of a teflon-like (C4F8) polymer to ensure hydrophobicity; to do this, a modified Bosch/RIE process was utilized (SI 500 Sentech Instruments Gmbh, Berlin, Germany), where solely the passivation mode was activated. In this phase, all gas flows, including SF6, argon, and oxygen, are set to zero, with the exception of the chemical inert passivation layer C4F8.

### SEM Characterization

Several SEM images of the samples were captured to assure reproducibility and repeatability, utilizing a Dual Beam (SEM-FIB) - FEI Nova 600 NanoLab system. During the acquisitions beam energies of 5 and 15 keV, and corresponding electron currents of 0.98 pA and 0.14 nA, were used. In some cases the mode 2 configuration was set, through which images can be magnified over 2.5 × 10^6^ times, and ultrahigh resolution can be achieved.

### Contact angle characterization

Surface hydrophobicity of the samples was determined by measuring the water contact angle with one drop of about 5 μl of D.I. water using an automatic contact angle meter (KSV CAM 101, KSV Instruments LTD, Helsinki, Finland) at room temperature. Four measurements were performed on each substrate to evaluate the average contact angle θ, at 5 s.

### Measuring the electrical activity of species in solution

Small drops (*V* < 10 μL) of D.I. water containing infinitesimal amounts of analytes were gently positioned upon the surfaces. In experiments where the concentration of those analytes was varied over a significant range, the ion current of the species in solution was measured demonstrating a good sensitivity of the device. Deionized (DI) water was used for all experiments. All chemicals, unless mentioned elsewhere, were of analytical grade and were used as received. The electrical response of biosensors has been measured using a 2-channel source/measure precision unit (Agilent B2902A), controlled by a home-made software. Gold metal thin film have been contacted through high precision metallic tips. Each chip contains 5 active points in a line and every measurements have been made contacting only one point at time. The two gold electrode were contacted on each side, in such a way to let the current go through the active PEDOT:PSS channel between the contacts, which represents source and drain in the transistor architecture. The contacts reach the top of one pillar, as described in the previous part, and the active channel in between results in contact with the liquid drop on the pillars. The drop of electrolyte has been suspended over the pillars, on top of the contacts on the surface in the central part of the chip. Ag wire, in the case of saline solutions and Pt wire in the case of Adrenaline and CTAB, are immersed into the electrolyte drop, acting as gate electrode (the schematic of the operative device is reported in the Supporting Information Figure #5.1). Biosensors devices were characterized by recording output characteristics (*I*ds versus *V*ds) of NaCl, at concentration of 1 × 10^−3^ M. The output curves as in Supporting Information Figure #1.1 have been acquired at *V*ds between 0 and −1 V, in steps of 0.1 V, for a fixed *V*gs varied in the range between 0 and 1 V, with steps of 0.2 V. Biosensor modulation measurements were acquired by measuring *I*ds versus time (Fig. 3) under a constant *V*ds = −0.05 V for each specie (saline or molecular), while pulsing *V*gs between 0 V and a positive value that was gradually increased from 0 V to 1 V with a step of 0.2 V, with a time interval of 60 s. The gate current (*I*gs) was simultaneously acquired during measurements. The drain voltage *V*ds induces hole drifting in the channel, generating the channel current *I*ds. When a positive gate voltage (*V*gs) is applied, cations (M^+^) from the electrolyte are forced to enter the PEDOT:PSS channel and dedope it according to Equation 4 as follows


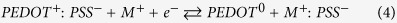


This acts as a dedoping process, because cations entering the polymer make the drain current module |*I*ds| decrease, due to the smaller number of holes available for conduction. On the contrary, when *V*gs = 0 V is applied, ion diffusion occurs from the PEDOT:PSS to the electrolyte, and the number of conducting holes increases. This is named a doping process, as in this case |*I*ds| increases. As a consequence, the drain current module |*I*ds| decreases when *V*gs > 0 V is applied and increases when *V*gs is set again to 0 V (Supporting Information Figure #1.1). Biosensor current response is expressed as current modulation ΔI/I_o_ = (I − I_o_)/I_o_, where *I* is the drain current value measured for *V*gs > 0 V and I0 is the *I*ds value for *V*gs = 0 V. For each measurement, *I* and *I*0 values were obtained from current transient measurements considering the quasi-steady state current values.

### Statistical Analysis

The current traces as in Fig. 3 and successive Figures have been acquired over at least 20 repetitions per gate potential, in which the repetitions have been realized in a short time interval and this guarantees that the experimental conditions are maintained constant (including the temperature and air humidity) over the whole duration of the experiment, that in turn assures reproducibility. Under these conditions, the amount of variation of values around the average is vanishingly small and thus negligible. Thus, the errors in measuring the currents have been calculated but are not represented in the diagrams because small respect to the average (e < 0.01). Pair-wise comparisons between means of different groups were performed using a Student’s *t*-test (two tailed, unpaired) where, for each couple of normally distributed populations, the null hypothesis that the means are equal was verified. Everywhere in the text the difference between two subsets of data is considered statistically significant if the Student’s *t*-test gives a significant level *P* (*P* value) less than 0.05.

### Solute distribution within the droplet

The distribution of a trace in a slowly evaporating droplet was derived on solving the Langevin equation^32^,^36^,^41^


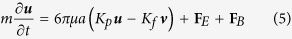


where **u** is the unknown velocity vector for the particle, **v** is the unperturbed fluid velocity, *a* is the particle radius, and m is the particle mass. The first term on the right-hand side of Eq. (5) represents the hydrodynamic drag on the particle. In Eq. (5), **F**_*E*_ = *zeE*_*x*_ ***e***_*x*_ represents the electrostatic force that is exerted by the externally applied Electric field, while 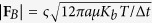 is the Brownian force. The significance of the additional terms in Equation (5) and variables therein is given as follows: *z* is the charge number, *e* ~ 1.610^−19^ *C* is the elementary charge, 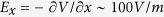 is the electric field that here has longitudinal component solely and is derived starting from the applied voltage *V*, assuming that *V* varies linearly along the droplet; *ς* is a Gaussian number with zero mean and unit variance; *μ* = 10^−3^ *Pa s* is the viscosity of water; *T* = 298 *K* is the temperature of the system; Δ*t* is the discrete time step of the simulation specified below. K_p_ and K_f_ are diagonal matrices describing the additional hydrodynamic hindrance associated with interactions between the particle and the system boundaries are here set equal to zero. For the unperturbed fluid velocity we used the solution provided by Tam and colleagues^29^, who developed an analytical solution to the thermo-capillary driven Marangoni flow in a small droplet of water sitting on a super-hydrophobic surface in terms of stream-functions. Details on the derivation and the description of the velocity profile within the droplet are provided in a separate Supporting Information file #2. Equation (5) was solved using a numerical scheme. The simulations are forward Euler integrations of the finite-difference equations resulting from discretization of the diffusion and convective operators as in^45^,^46^. The initial mesh consists of N = 400 grid points. The time step used is Δt = 10^−3^ s. Initially, the entire system was placed in the initial condition, where p = 500 identical particles are distributed uniformly within the domain. The system was then integrated for 6,000 time steps and images were saved at specific time points. In all cases, the initial disturbance propagated outward from the initial position to the border of the drop. We found that the radial distribution of solute inside the drop depends on size and charge of the dislodged particles and this is described in the Results of the paper. In solving the equations, at the boundaries Dirichlet of fixed conditions are imposed, and thus the concentration of particles Ψ is set as zero at any time *t*, Ψ(*t*, *R*) = 0: this implies that the analysis is valid for the time that the initial perturbation takes to spread over the entire grid. The propagation of the solute was found to depend on the convective flow within the droplet, with the leading edge of the perturbation moving unsteadily with time. The propagation velocity of the initial perturbation and thus the dynamic response of the system depends on the parameter values.

## Additional Information

**How to cite this article**: Gentile, F. *et al.* Geometrical Patterning of Super-Hydrophobic Biosensing Transistors Enables Space and Time Resolved Analysis of Biological Mixtures. *Sci. Rep.* **6**, 18992; doi: 10.1038/srep18992 (2016).

## Supplementary Material

Supplementary Information

## Figures and Tables

**Figure 1:**
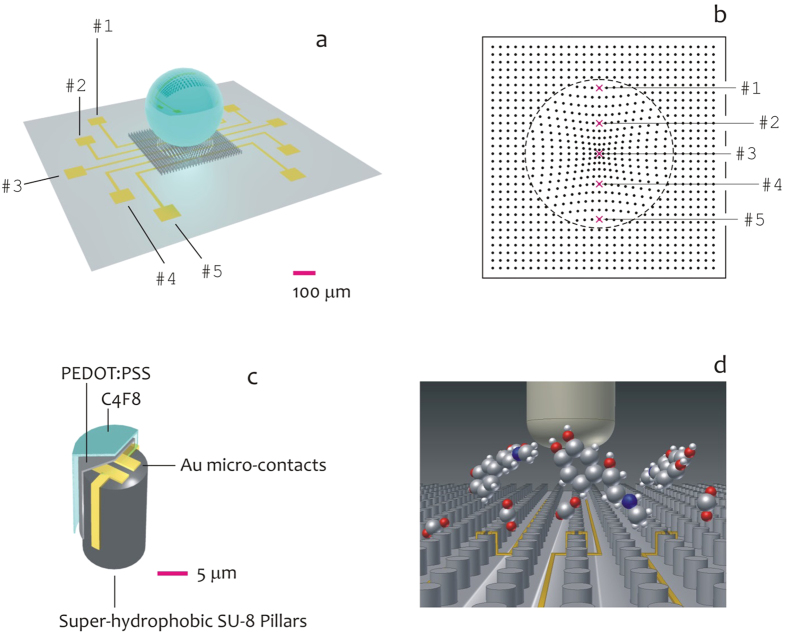
Artistic representation of the super-hydrophobic device and the silicon substrate on which micro electrodes are patterned for site selective analysis of a solution (a); top view of the pattern of super-hydrophobic pillars, the pattern is non periodic and the pillar to pillar distance smoothly transitions from a maximum, at the periphery of the pattern, to a minimum at the center of the pattern, this would automatically center the drop of a solution with the super-hydrophobic pattern (**b**); a finite number of super-hydrophobic pillars in the pattern are tailored with Au micro contacts, and are covered with PEDOT:PSS for electrical measurement of the solute and *C*_4_*F*_8_ that assures hydrophobicity to the whole structure (**c**); the cartoon reproduces a pattern of super-hydrophobic pillars on the top of which a solution of CTAB and adrenaline is deposited. The device operates the separation of molecules in a super-hydrophobic drop based on their size and charge and thus may resolve biological mixtures in a solution (**d**).
