# Supplementary material for: Geometrical Patterning of Super-Hydrophobic Biosensing Transistors Enables Space and Time Resolved Analysis of Biological Mixtures
Source: Sci Rep. 2016 Jan 12;6:18992. doi: 10.1038/srep18992 (PMC4709515; doi:10.1038/srep18992)
Supplement: Supplementary Information [file srep18992-s1.docx]

**Supporting Information**

**Geometrical Patterning of Super-Hydrophobic Biosensing Transistors Enables Space and Time Resolved Analysis of Biological Mixtures**

Francesco Gentile^1,2^, Lorenzo Ferrara^3^, Marco Villani^4^, Manuele Bettelli^4^, Salvatore Iannotta^4^, Andrea Zappettini^4^, Mario Cesarelli^1^, Enzo Di Fabrizio^5^, Nicola Coppedè^4†^

^1^ Department of Electrical Engineering and Information Technology, University of Naples, 80125, Naples, Italy

^2^ Department of Experimental and Clinical Medicine, University of Magna Graecia, 88100 Catanzaro, Italy

^3^ Istituto Italiano di Tecnologia, Via Morego 30, 16163 Genova, Italy

^4^ IMEM-CNR Parco Area delle Scienze 37/A - 43124 Parma, Italy,

^5^ King Abdullah University of Science and Technology, Thuwal 23955-6900, Kingdom of Saudi Arabia

**File #1. Measuring salts in solution**.


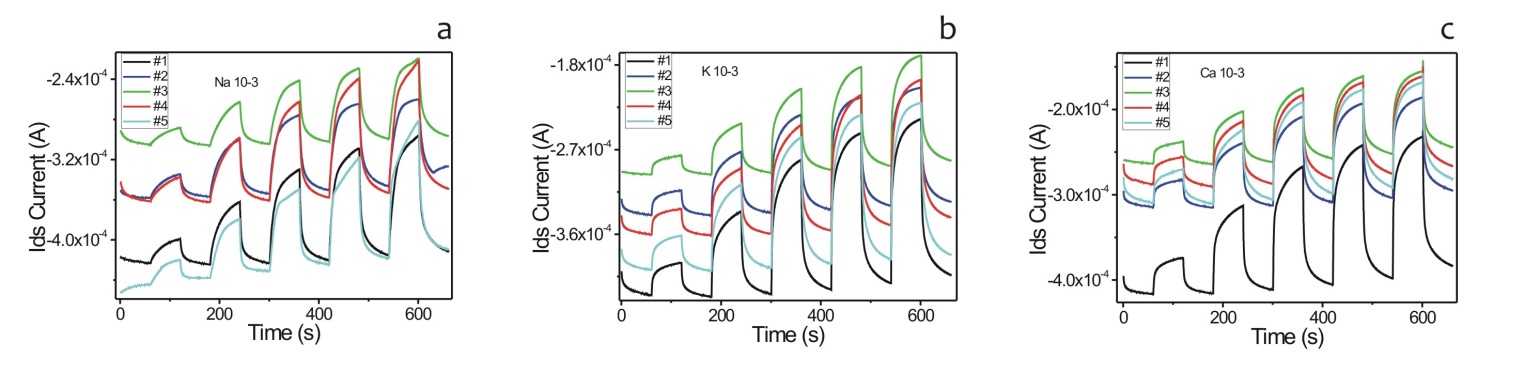


**Supporting Information Figure #1.1**


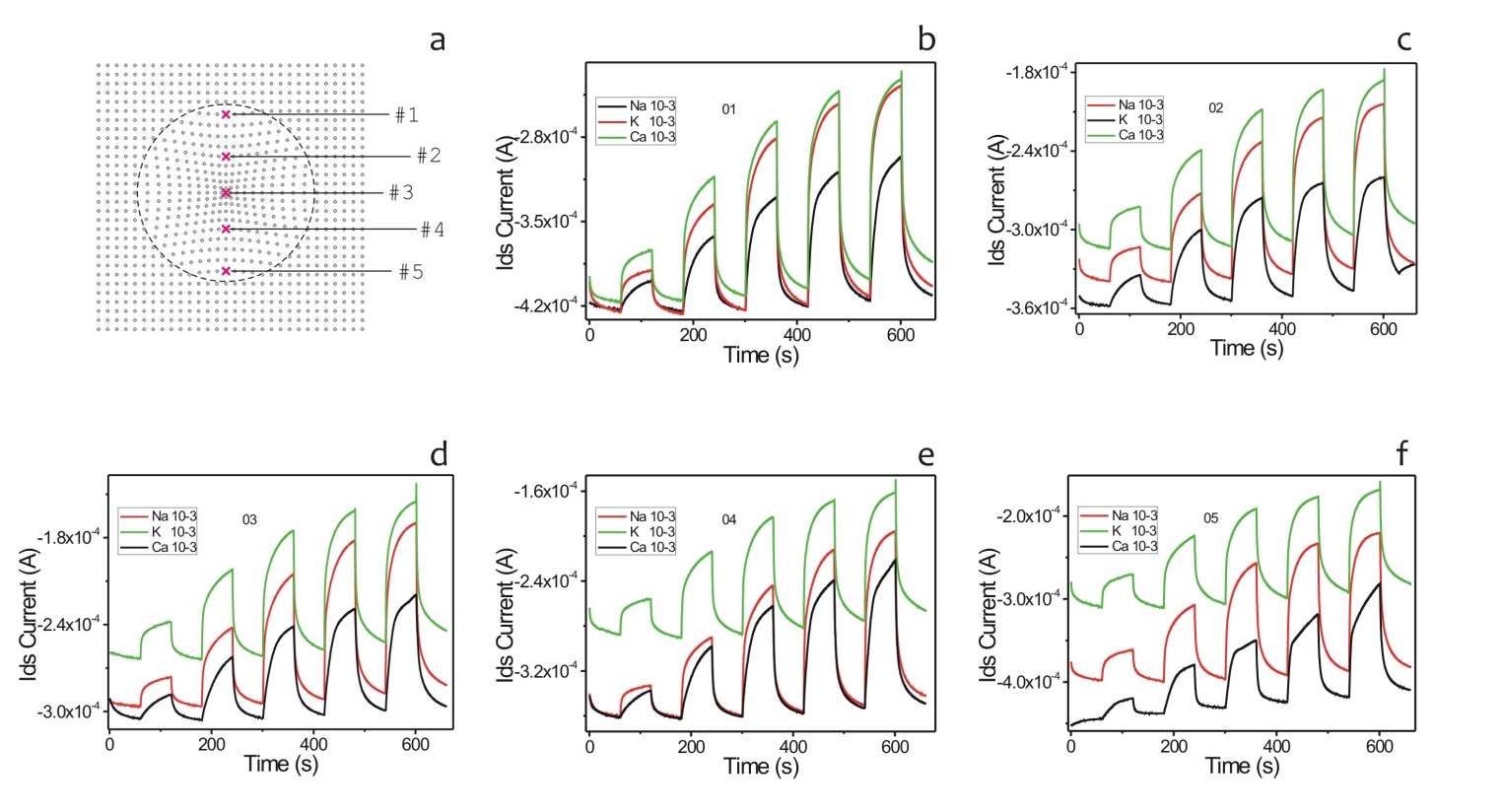


**Supporting Information Figure #1.2**


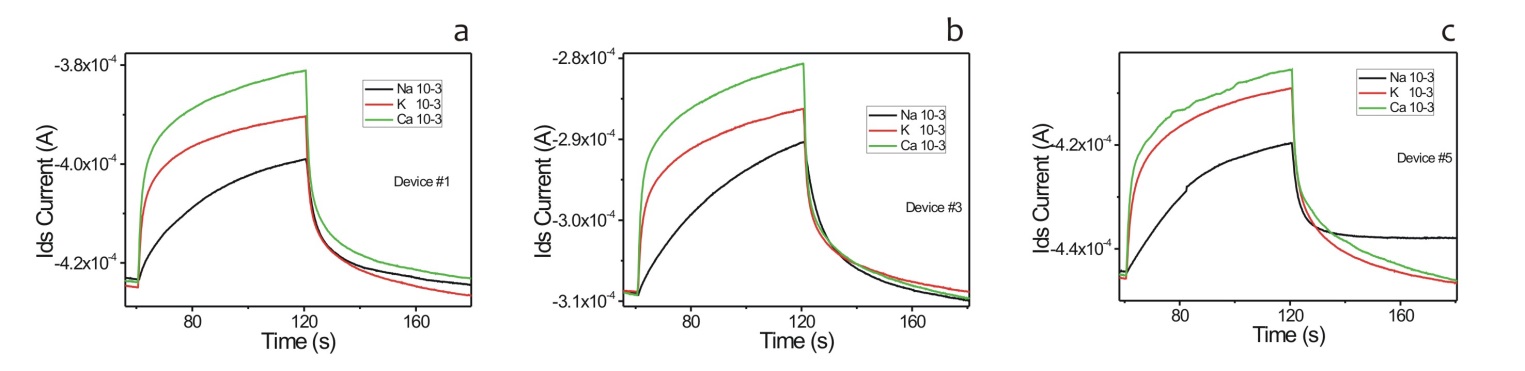


**Supporting Information Figure #1.3**

**Supporting Information File #2. Solute distribution within the droplet**. The distribution of a trace in a slowly evaporating droplet is derived on solving the Langevin equation ^1-3^

| $m\frac{\partial\boldsymbol{u}}{\partial t}=6\pi\mu a\left( K_{p}\boldsymbol{u}-K_{f}\boldsymbol{v} \right)+\mathbf{F}_{E}+\mathbf{F}_{B}$ | (1) |
| --- | --- |

where **u** is the unknown velocity vector for the particle, **v** is the unperturbed fluid velocity, and all the remaining terms in (1) are defined in the Methods of the article. For the unperturbed fluid velocity we used here the solution provided by Tam and colleagues^4^, who developed an analytical solution to the thermo-capillary driven Marangoni flow in a small droplet of water sitting on a super-hydrophobic surface in terms of stream-functions $\psi\left( r,\theta\right)$

| $\psi\left( r,\theta\right)=-\frac{1}{8}\left( 1-r^{2} \right)\left[ 1+r cos\theta-\frac{1-r^{2}}{\left( r^{2}+1-2r cos\theta\right)^{\frac{1}{2}}}+\sum_{n=2}^{\infty} \frac{\left( n-1 \right)-2\left( n-1 \right)Bi}{\left( 2n-1 \right)\left( \left( n-1 \right)+Bi \right)}r^{n}\left( P_{n-2} cos\theta-P_{n} cos\theta\right) \right]$, | (2) |
| --- | --- |

in which $r$ and $\theta$ are the non-dimensional polar coordinates as in the frame of reference in the **supporting information figure #2.1**, $Bi$ is the Biot number, $P_{n}$ is the Legendre polynomial of order $n$.


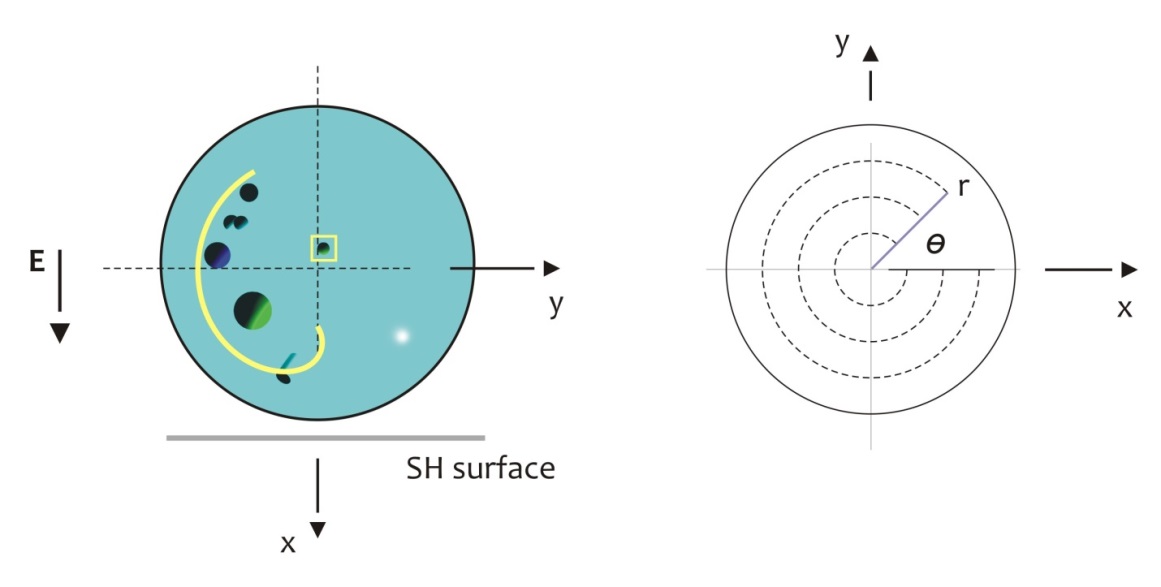


**supporting information figure #2.1**

The velocity field can be derived from the stream-functions being

| $v_{r}=-\frac{1}{r^{2}sin\theta}\frac{\partial\psi}{\partial\theta}$,  $v_{\theta}=\frac{1}{r sin\theta}\frac{\partial\psi}{\partial r}$, | (3) |
| --- | --- |

which is rephrased in rectangular coordinates as

| $v_{x}=v_{r} cos\theta+r v_{\theta} sin\theta$,  $v_{y}=v_{r} sin\theta-r v_{\theta} cos\theta$. | (4) |
| --- | --- |

The stream-lines for the present configuration were derived and are reported in the form of a contour plot in the **supporting information figure #2.2**. The rectangular components of the velocity field $v_{x}$ and $v_{y}$ were accordingly determined using Equations 3-4 and are displayed in the diagrams in the **supporting information figure #2.3**.


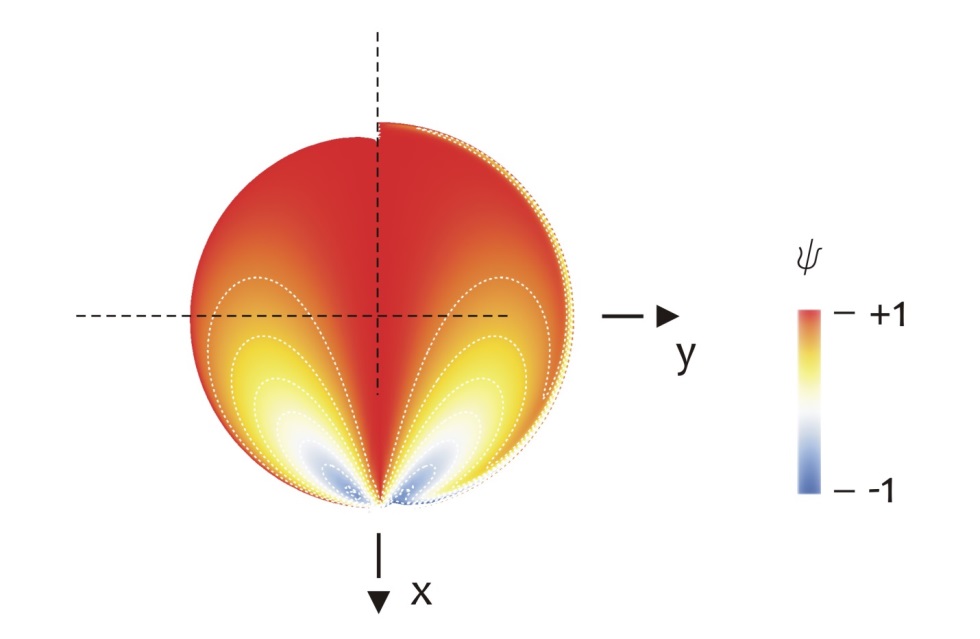


**supporting information figure #2.2**


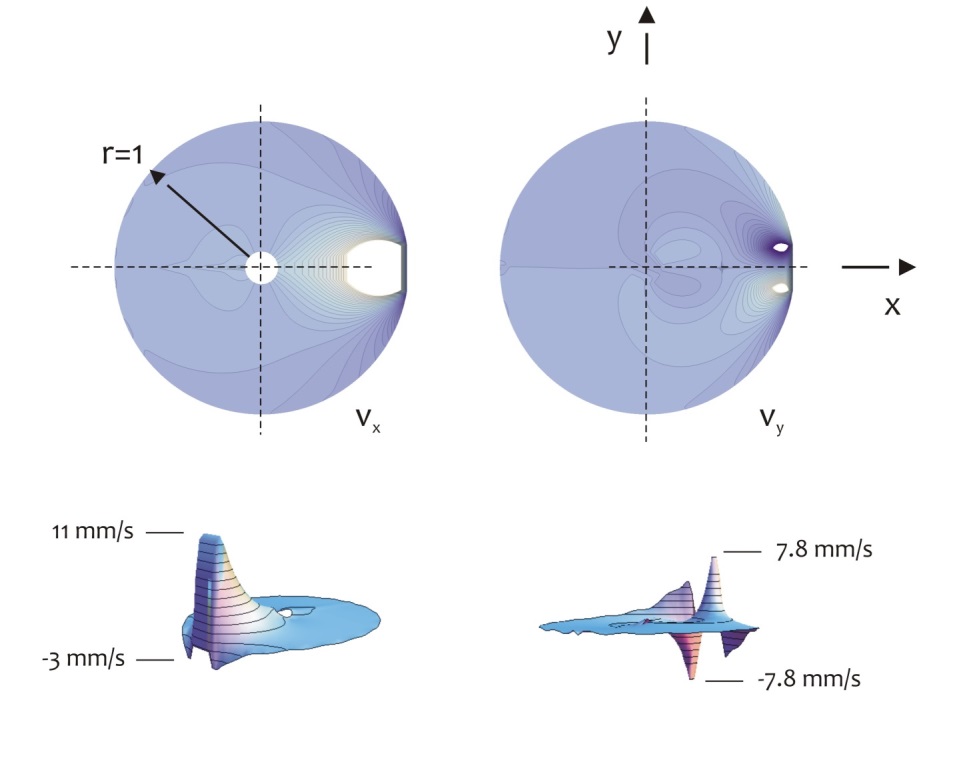


**supporting information figure #2.3**

From the diagrams in the **supporting information figure #2.3**, the corresponding vector velocity field may be reconstructed and this is reported in the **supporting information figure #2.4**.


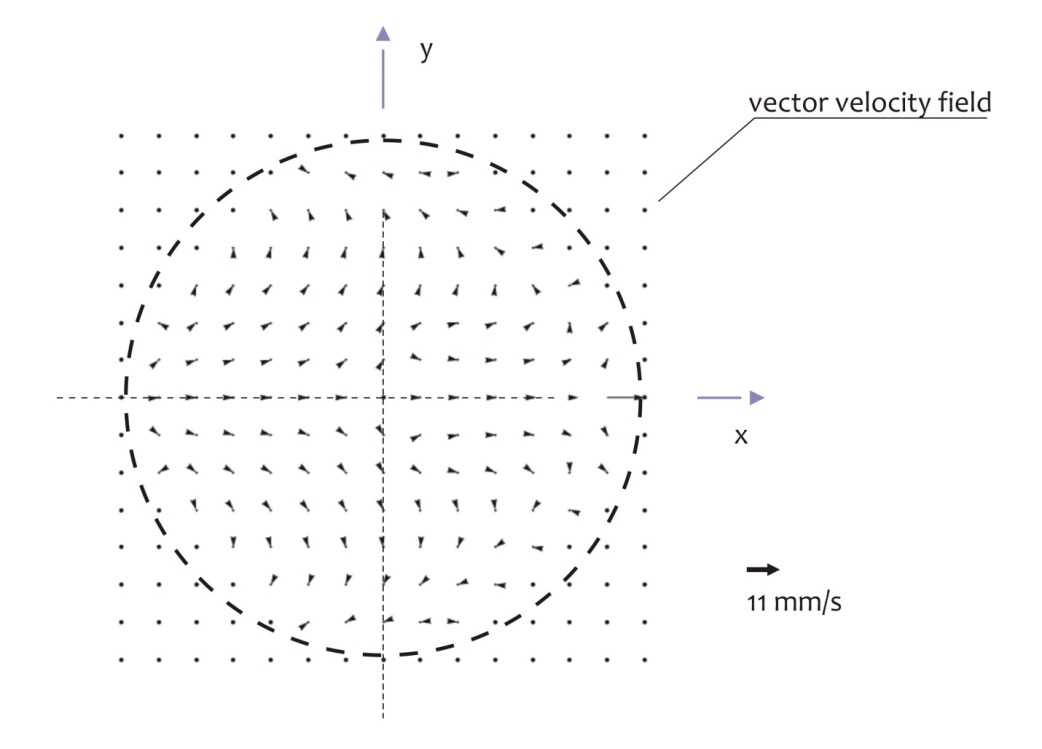


**supporting information figure #2.4**

On solving Equation (1) in which the correct unperturbed velocity profile has been substituted (see also the Methods of the paper) we derived the radial distribution of solute (that is, an ensemble of 500 particles) inside the drop on time. Examples of this distribution are reported in the **supporting information figure #2.5** for the time parameter $t$ ranging from $t=0 s$ to $t=0.5 s$ and the for the particle size $a=2 nm$. We found that the radial distribution of solute inside the drop depends on size and charge of the dislodged particles and this is described in the main text of the paper.


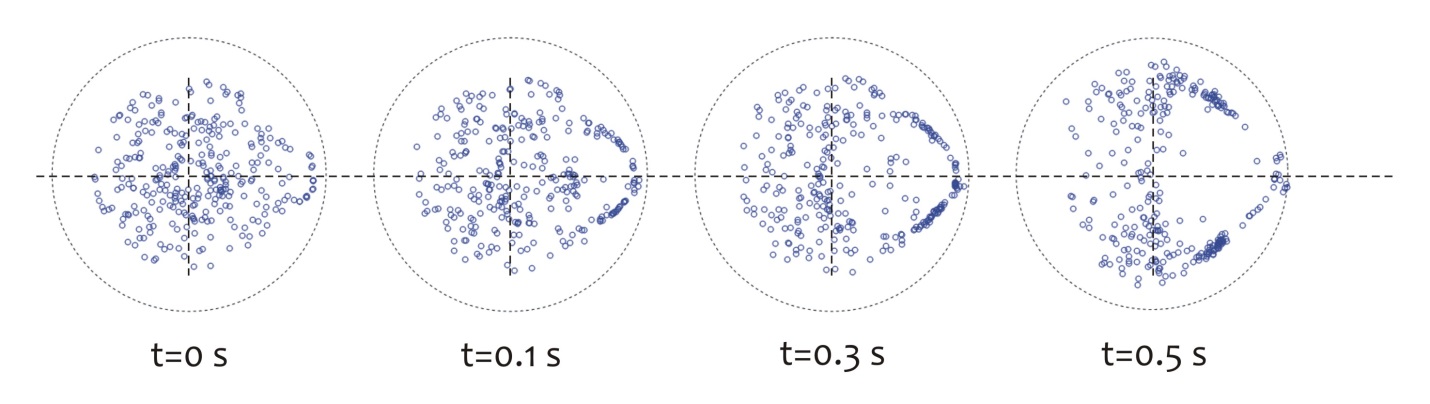


**supporting information figure #2.5**

**Supporting Information #3.** **Schematic representation of the devices**. **Supporting Information Figure #3.1**, is an artistic representation of the devices, which are given by the super-position of two layers, and this is described in the Methods of the Article. Layer A is the silicon substrate with the gold conductive circuits, differently, Layer B is the variable lattice of super-hydrophobic micro pillars which permit solution manipulation (**Supporting Information Figure #3.2a-b**). In the lattice, five pillars incorporate micro-electrodes for space-resolved measurements of biological mixtures in a super-hydrophobic drop (**Supporting Information Figure #3.2c - Supporting Information Figure #3.3**).


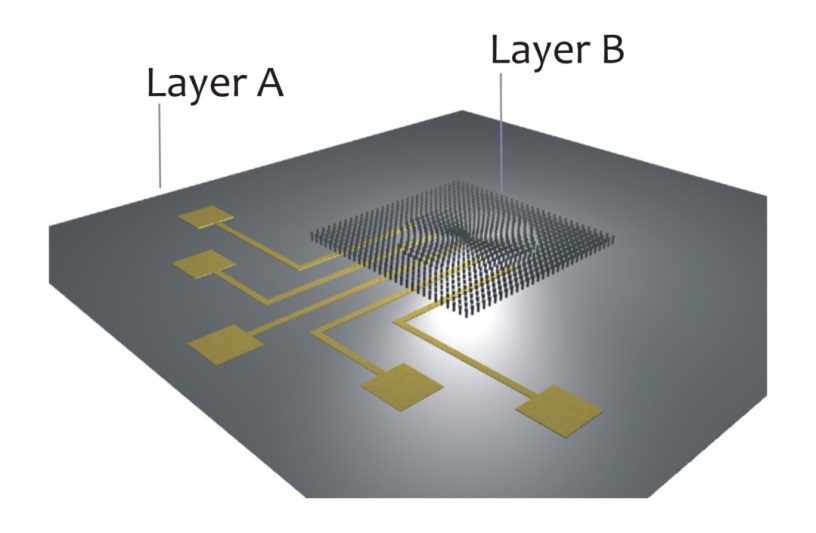


**Supporting Information Figure #3.1**


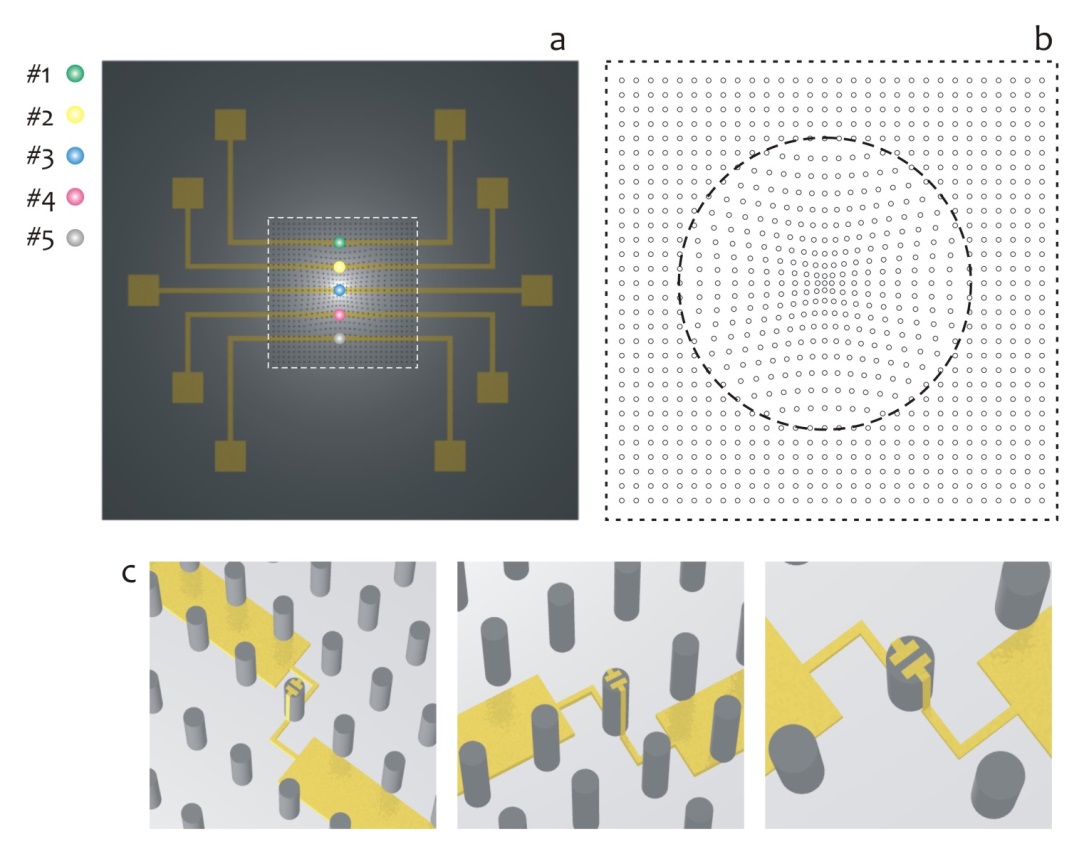


**Supporting Information Figure #3.2**


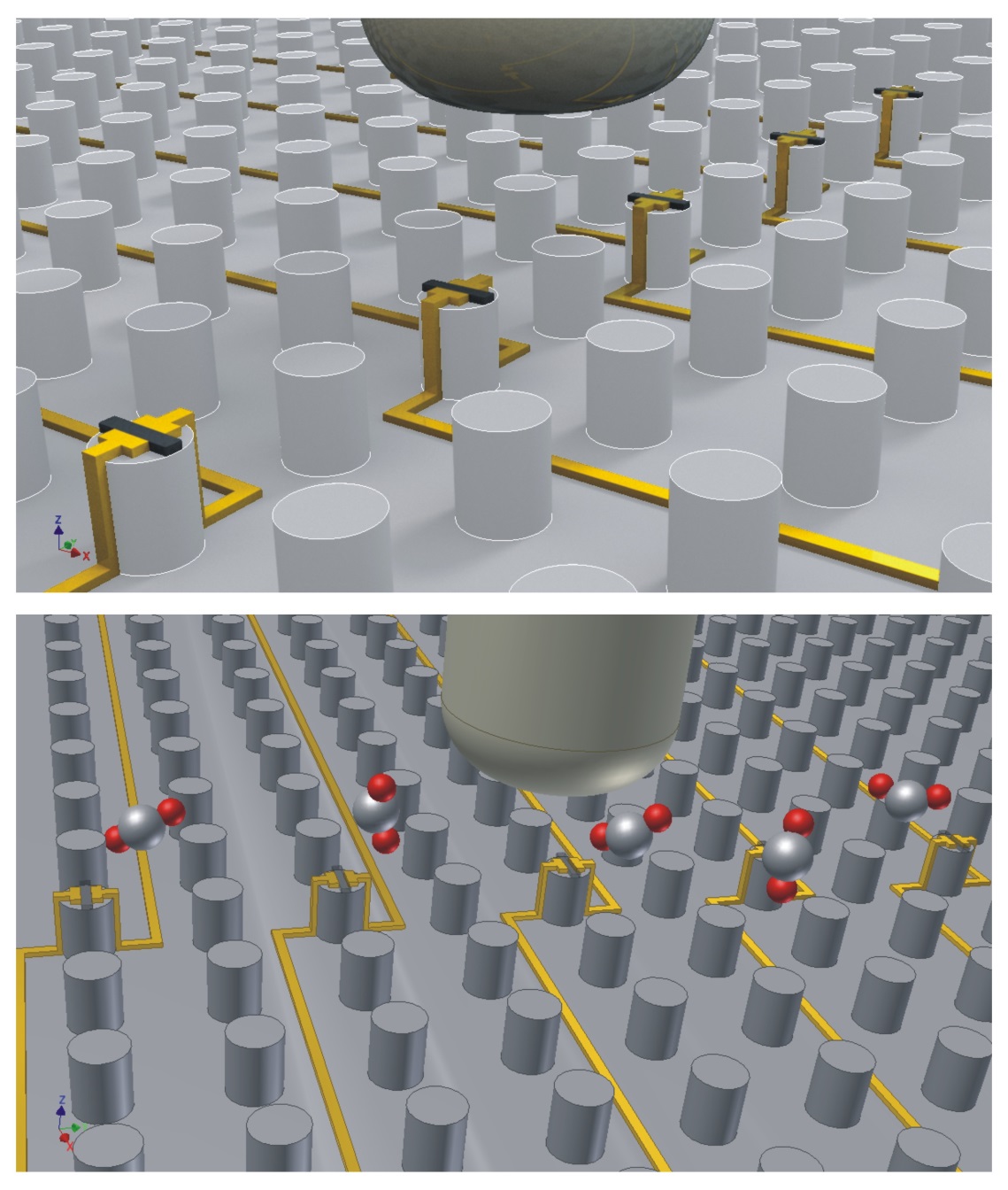


**Supporting Information Figure #3.3**

**Supporting Information #4 Detailed Description of the Fabrication Process**. Here, we provide further details on the process of fabrication of the device. **Supporting Information Figure #4.1** is a schematic of the device fabrication. From left to right, the Si substrate is covered by a layer of PMMA, subsequently exposed to the UV while shielded by the first mask. The deposition of gold and the lift off of the PMMA creates the final gold connection. Then a layer of SU8 is deposited and exposed to the UV shielded by the second mask. The development of the SU8 removes the unexposed layer, creating the pillars.


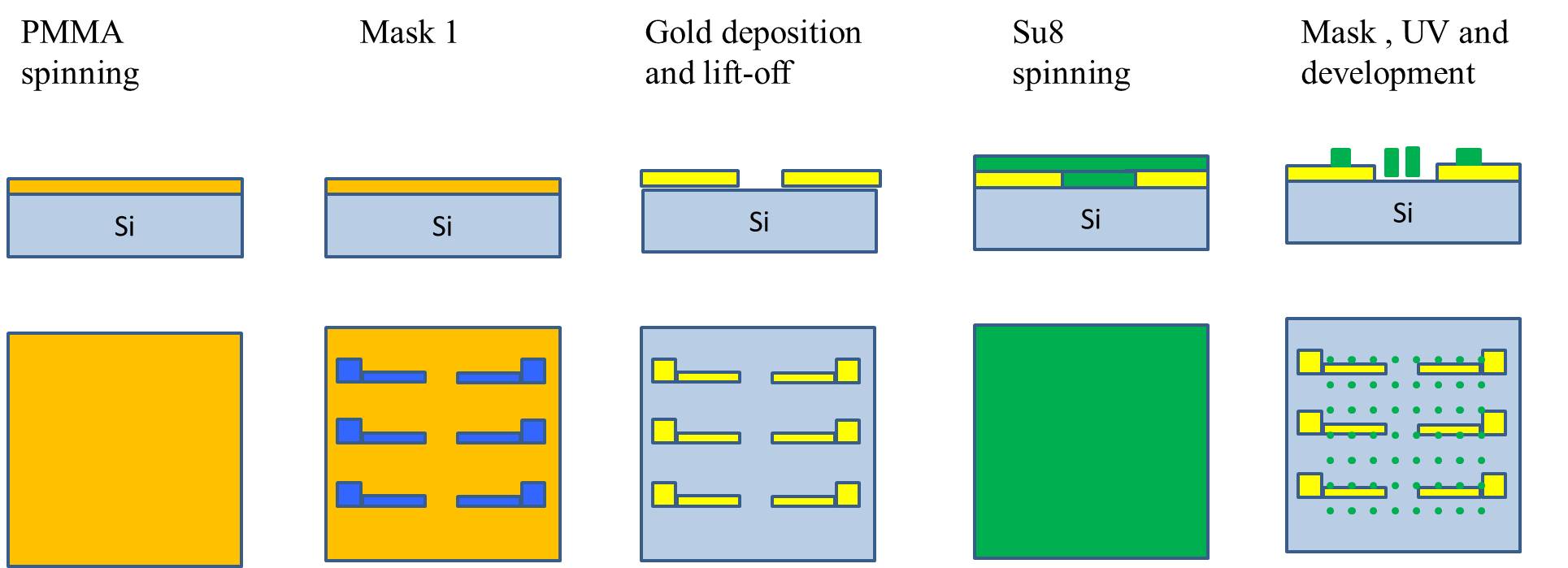


**Supporting Information Figure #4.1**

A limited number (in the present work, 5) of super-hydrophobic SU8 pillars are further modified to incorporate micro-electrodes, and this is described in the Methods of the main article and in the **Supporting Information Figure #4.2**.


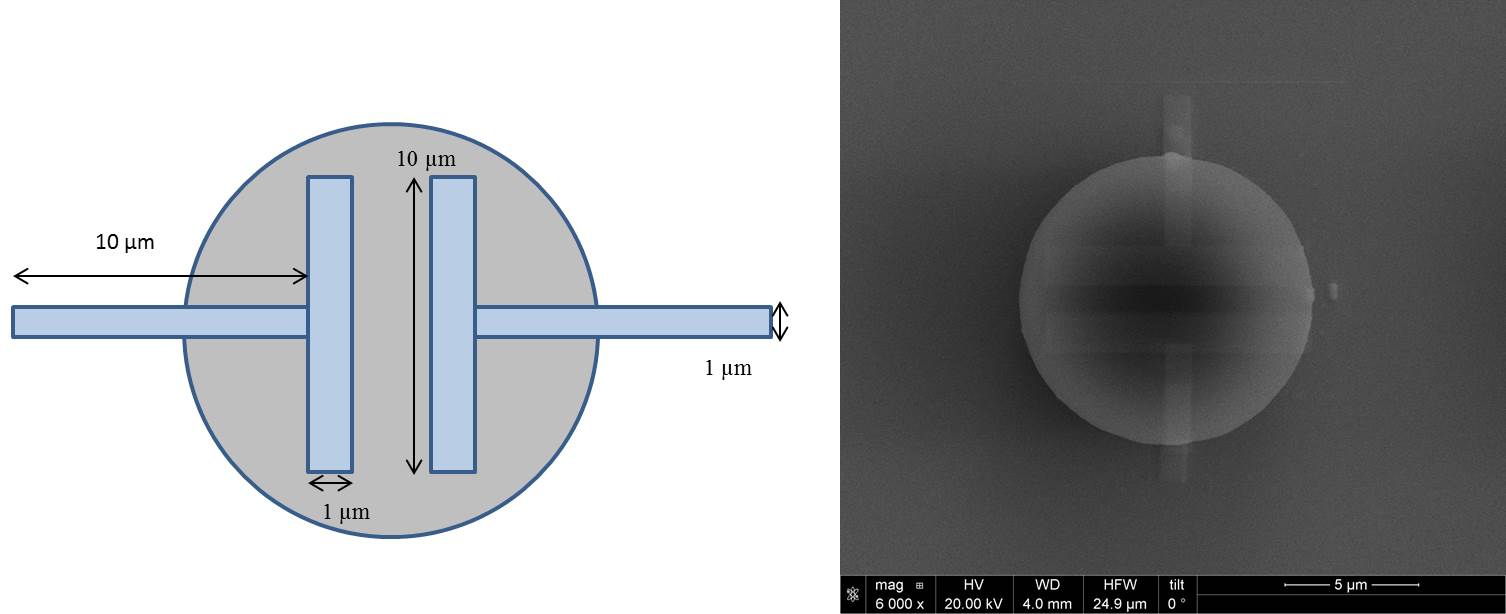


**Supporting Information Figure #4.2**

The schematic of the masks used for the realization of Layer A and Layer B is reported in the **Supporting Information Figure #4.3**.


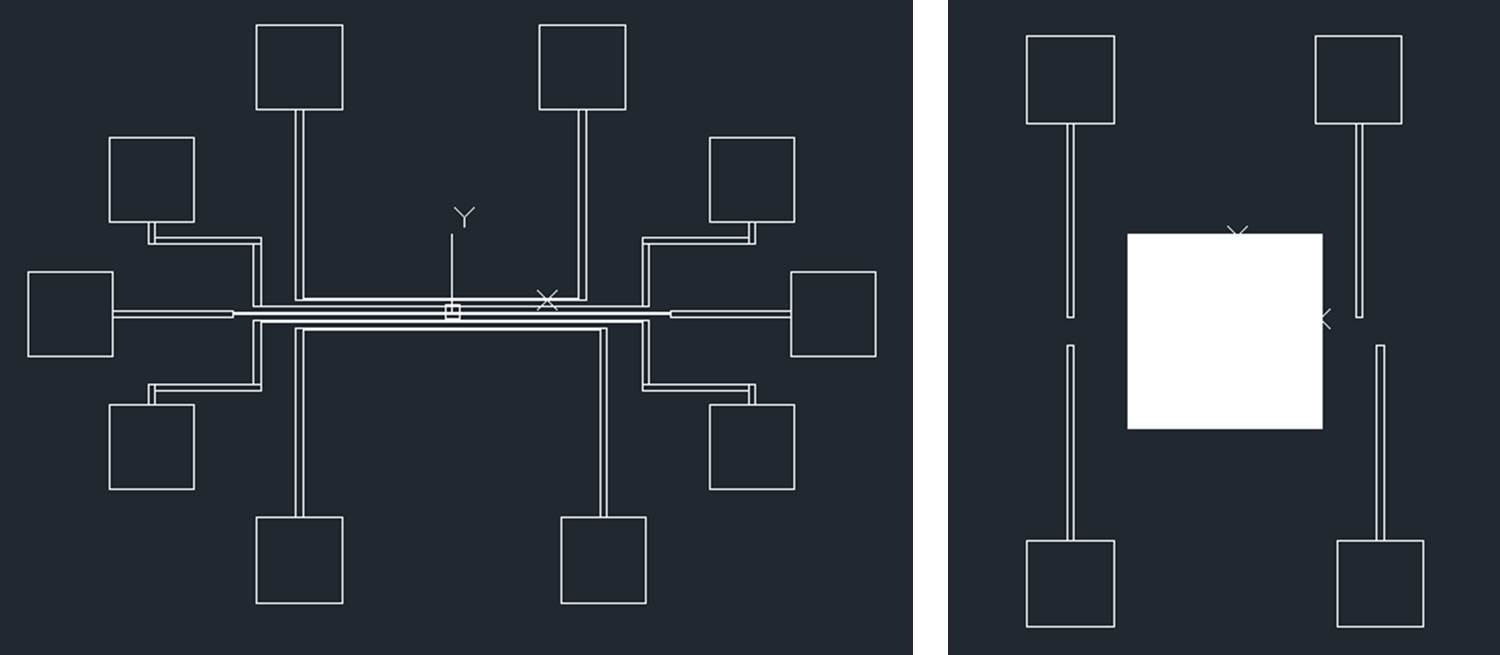


**Supporting Information Figure #4.3**

The CAD layout of the first masks consists in 10 square contacts areas of 4 mm^2^, connected with lines with 200 µm width in the initial region and 30 µm in the last, horizontal region. The 5 lines at the left side are separated from the 5 lines at the right side by a gap of around 60 µm. The layout of the second mask consists in the replication of 4 square contacts areas and connections for the purpose of alignment respect to the previous mask, plus a central area of 25 mm^2^ filled with circles of 10 µm diameter. As shown in the **Supporting Information Figure #4.4**, the circles have a perpendicular arrangement, with a pitch of 30 µm center to center, but in the central part of the array they assume a radial arrangement, becoming denser towards the center and this is described in the methods of the article.


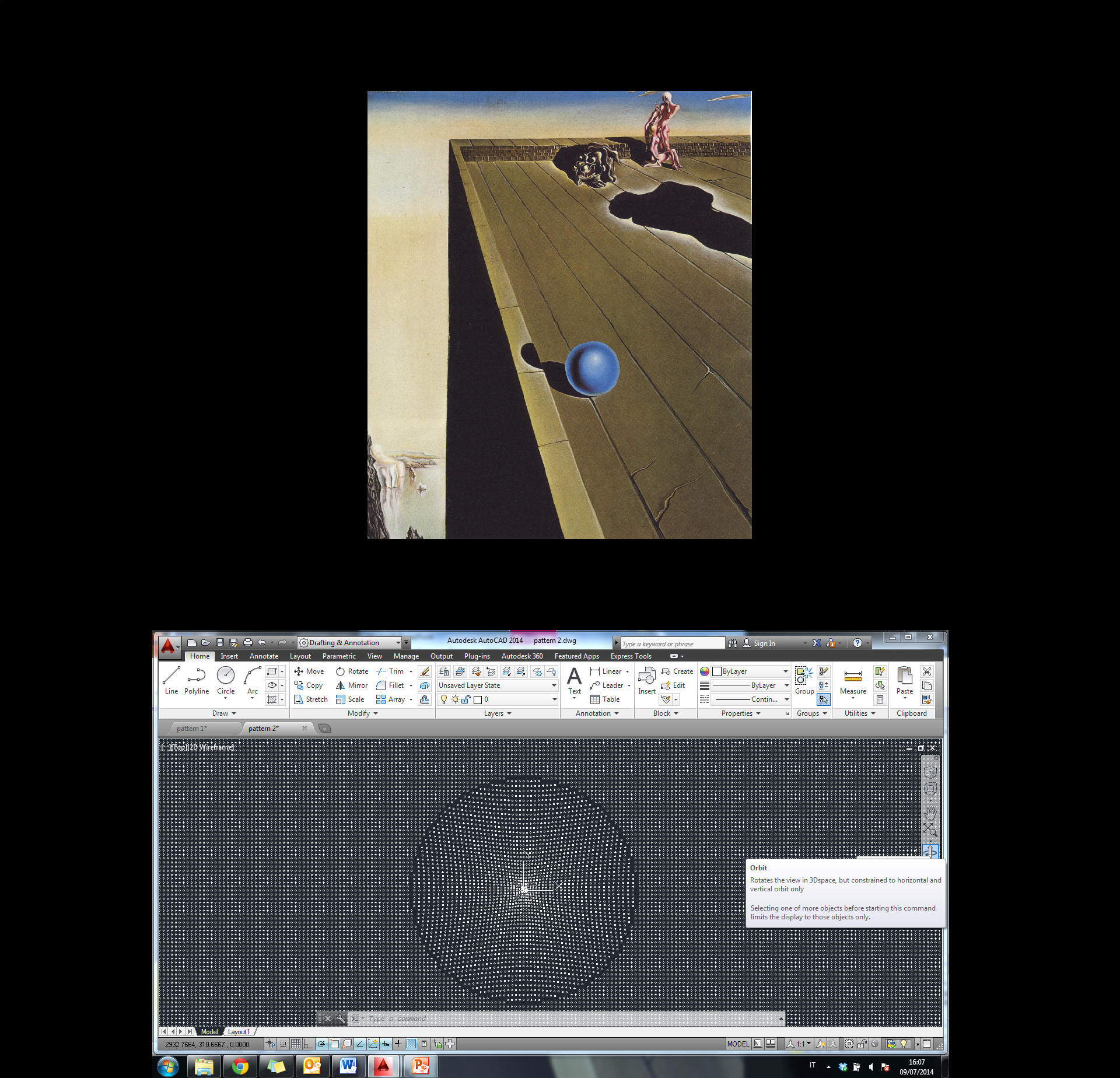


**Supporting Information Figure #4.4**

For the first mask solely, the layout is elaborated and processed by a NPGS (Nanometer Pattern Generation System) software that convert the CAD layout into patterns for SEM (Scanning Electron Microscope) lithography. In this way, each geometrical object is defined with an electron beam exposure time, dose and pitch. We choose an electronic beam current of 1.8 nA with 50 nm pitch for the circles definition, while we choose a current of 6.4 nA and 100 nm pitch for the contact definition.

For the second mask, direct laser writing was used (Heidelberg DWL 66fs), setting the parameters used already in^5^.

The two masks are prepared starting from conventional photomask blanks, made by a substrate of quartz with a thin layer of chrome on one surface and a photosensitive layer on top. When inserted in the SEM main chamber, the electron beam exposes the areas defined by the initial CAD design with the abovementioned parameters. Once the exposure has ended, the photomask is developed, so that the exposed part is removed. The mask is treated with a solution of chrome etch, to remove the chrome only in the patterned area. Once this process is concluded, the photoresist is completely removed and the mask is complete.

**Supporting Information #5.** **Operating the device**. In the following, we present a 3D representation of the device, in which source, gain and gate are clearly evidenced for ease of visualization.


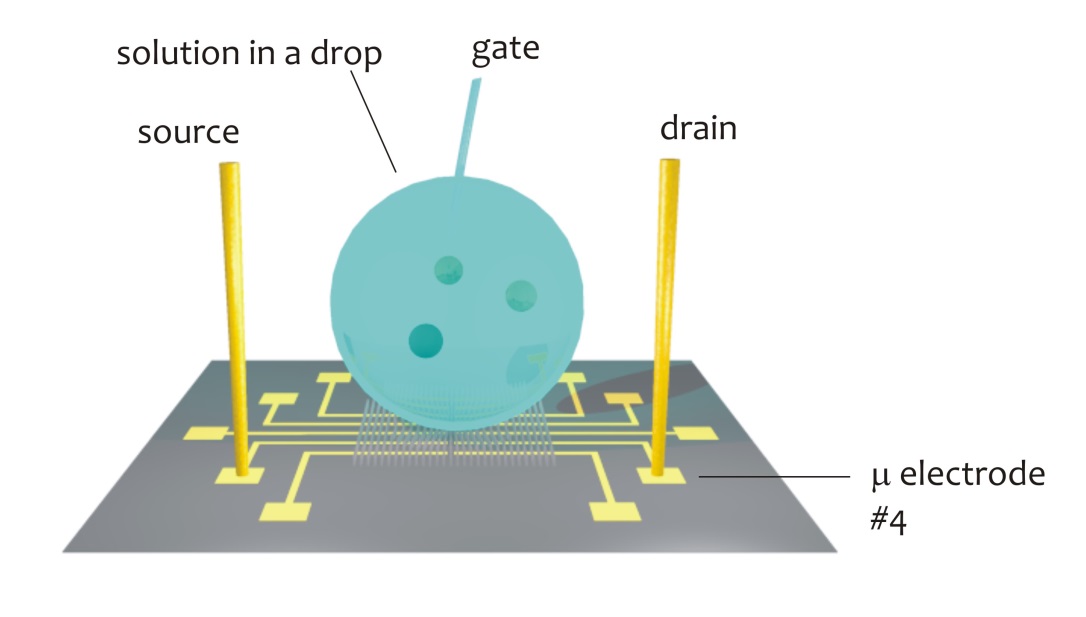


**Supporting Information Figure #5.1**

**References**

1 Astier, Y. *et al.* Artificial Surface-Modified Si3N4 Nanopores for Single Surface-Modified Gold Nanoparticle Scanning. *Small* **7**, 455-459 (2010).

2 Kim, M.-m. & Zydney, A. L. Effect of electrostatic, hydrodynamic, and Brownian forces on particle trajectories and sieving in normal flow filtration. *Journal of Colloid and Interface Science* **269**, 425–431 (2004).

3 Gentile, F. *et al.* Selective on site separation and detection of molecules in diluted solutions with superhydrophobic clusters of plasmonic nanoparticles. *Nanoscale* **6**, 8208-8225 (2014).

4 Tam, D., von Arnim, V., McKinley, G. H. & Hosoi, A. E. Marangoni convection in droplets on superhydrophobic surfaces. *Journal of Fluid Mechanics* **624**, 101- 123 (2009).

5 Gentile, F. *et al.* Direct Imaging of DNA Fibers: The Visage of Double Helix. *Nano Letters* **12**, 6453−6458 (2012).
